# Experimental validation and simulation of a U-Shaped elastic beam robot for stable running locomotion

**DOI:** 10.1038/s41598-025-23496-9

**Published:** 2025-12-01

**Authors:** Wael Khalifa, Mahmoud A. Essam, M. Riad Ghazy, Ahmed Abu El-fadl

**Affiliations:** 1 Mechanical Engineering Department, Higher Technological Institute, Tenth of Ramadan, P. O. Box: 228, Egypt; 2 Faculty of Engineering, Zagazig University, Zagazig city, 44519 Egypt

**Keywords:** Legged locomotion, Vibration-driven robot, U-shaped elastic beam, Experimental validation, Torsional vibration dynamics, Engineering, Physics

## Abstract

This paper presents an innovative terrestrial robot employing a vibration-based locomotion system powered by a single DC motor with an eccentric rotating mass. The robot, composed of a U-shaped aluminum elastic beam and lightweight wooden feet, attains steady mobility by synchronizing torsional vibrations with centrifugal forces. Experimental and simulated analyses were performed at three angular velocities (ω_1_ = 125.66 rad/s, ω_2_ = 251.33 rad/s, ω_3_ = 376.99 rad/s) to assess stability, deviation, velocity, and hopping performance. The results indicated that a rise in angular velocity significantly improved locomotion efficiency. At ω_1_, the robot attained an average body velocity of 85.46 mm/s and a hopping distance of 405.88 mm. At ω_2_, the velocity rose to 221.24 mm/s with a hopping distance of 418.24 mm, but ω_3_ achieved optimal performance with a velocity of 265.49 mm/s and a hopping distance of 424.08 mm. Deviation responses settled within ± 2 mm after 2.5 s at ω_3_, in contrast to more pronounced oscillations at ω_1_. Simulation results largely aligned with experimental outcomes in hopping distance (error < 3 mm at ω_3_) but routinely underestimated body velocity by 50–60%. The findings corroborate the suggested model for predicting vertical displacement, while underscoring the necessity for refinement to accurately capture horizontal velocity dynamics.

## Introduction

Vibration-based locomotion has emerged as a promising mechanism for propelling robots with minimal mechanical complexity, offering new possibilities for miniature, low-cost, and untethered robotic systems. Unlike traditional wheeled or legged robots that rely on complex actuators and control schemes, vibration-driven robots achieve motion through oscillatory inputs typically from motors with eccentric masses combined with asymmetric friction or structural compliance. This locomotion strategy is especially attractive in environments that demand simplicity, scalability, or high robustness, such as in confined spaces, swarms, or inside pipelines. The key component for adaptive running locomotion is use of passive dynamics and morphological computation^1^. Recently, a new approach has been proposed which uses passivity-based free vibration of curved elastic beams at resonance frequencies for stable legged locomotion^2,3^. In this approach, the main body of the robot is composed of elastic curved beam, and it is actuated by a single motor which oscillates a set of rotating masses. The centripetal force generated by these oscillating masses induces torsional or longitudinal vibration depending on the resonance frequency of robot structure. Related studies on structural vibration dynamics, including free elastic plates in orbital conditions^4^ and cracked cantilevered beams under harmonic loads^5^, further underline the importance of coupled vibration phenomena in understanding and optimizing resonance-based locomotion.

The foundational concept of using vibratory actuation for motion dates back to early prototypes of bristlebots, which use vibrating motors to generate directional movement through angled bristles. Cicconofri et al.^6^ provided a rigorous analysis of such systems, demonstrating how motion direction and speed can be controlled through frequency modulation and structural parameters. Similarly, Notomista et al.^7^ expanded this approach by introducing a unified modeling and control framework for vibration-driven robots, known as brushbots, capable of complex swarm behaviors without requiring traditional mobility hardware.

More advanced implementations have explored internal oscillators and anisotropic friction for planar motion. Zhan, Xu, and Fang^8^ developed a shell-like robot with two oscillators that achieved controllable steering by tuning the vibration amplitude and frequency, effectively manipulating inertial and frictional forces. Liu et al.^9^ introduced a vibration-powered cubic robot capable of stable and rapid locomotion on granular terrains, a significant advancement for soft or deformable surface navigation. Nguyen & Lab^10^ improved capsule robots via nonlinear elastic springs and oscillating masses resonant dynamics in enclosed geometries akin to U-beam systems.

In industrial inspection applications, especially in pipe networks, vibration-driven mechanisms have enabled compact robotic systems to move efficiently within narrow environments. Korendiy, V., et al.^11^ Proposed a solenoid-actuated in-pipe robot that employed a spring-lock mechanism to synchronize vibrations with pipe dimensions, achieving notable speeds despite a minimalist design. Korendiy, V. and O. Kachur^12^ modeled vibration-driven in-pipe robots with unbalanced internal masses and wheels; their methods emphasize inertial excitation and resonance similar to U-beam locomotion principles. V Gurskyi et al.^13^ studied the motion of a wheeled vibration-driven platform with a V-shaped spring-damper suspension and a centrifugal exciter. It introduces a generalized mathematical model to optimize design parameters for vibration-based systems used in pipeline inspection and cleaning.

Vibration-based locomotion, in which robots exploit internal oscillations and asymmetric surface interactions to move, has emerged as a compelling alternative to traditional wheel or leg driven mechanisms, especially at small scales and in constrained environments. These robots typically rely on simple vibrating actuators such as eccentric rotors, solenoids, or piezo-electrics and directional friction or anisotropic contact surfaces for propulsion^14,15^. In particular, U‑shaped visco‑elastic beams actuated near their torsional resonance can self‑organize into bounding locomotion without requiring sensory feedback or complex control architectures. A prominent early example deploys a simple rotating mass on a U‑shaped beam, demonstrating self-propelled bounding gait reminiscent of quadrupedal animals when driven at natural frequencies^14,16^.

For instance, hopping mechanisms based on curved beams exploit resonance to generate high payload-capable leaps^17^. While many systems rely on piezo-ceramic or ultrasonic vibration to power micro-robots and energy harvesters, their beam geometries sometimes L-shaped, sometimes U-shaped have been compared in terms of vibrational modes and efficiency^18^.

Moreover, investigations of symmetry-breaking in flexible hub-beam systems^19^ provide deeper insight into how geometric asymmetry and eccentric excitation influence locomotion performance, reinforcing the relevance of nonlinear beam dynamics in vibration-driven robot design.

Our surrounding terrains are unstructured, harsh, unstable, deformable, and unsafe. In such situations, legged robots have intrinsic movement benefits. They can cross terrain and hurdle barriers, making them ideal for various tasks. These include search and rescue, inspection in crowded and complex areas, planetary exploration, manufacturing, and agriculture^20^. In recent years, numerous legged systems have moved precisely and consistently in controlled environments^21^. However, there are still many obstacles to overcome before they can be widely used in everyday life^22^. They struggle in uncontrolled conditions despite excelling in controlled environments.

The prominent challenges of legged robot locomotion control are to locate appropriate places for creating foot contact with the environment and to produce corresponding dynamic movements.

Even state-of-the-art legged robots face challenges in adapting to varying terrains, slippery surfaces, negotiating over ground obstacles, managing payload and weight distribution, and recovering from stumbles^23,24^. Also, model predictive control (MPC) methods and numerical trajectory optimization are currently extensively utilized in the broad approach that pervades nearly all forms of legged robot locomotion. The same techniques are being modified for different leg designs and numbers of legs as well as for both flat ground and rough terrain. The energy efficiency is also much inferior compared to that of biological systems. The majority of research on robot locomotion on uneven terrain has centred on software development, including perception approaches. As a result, there is a lack of assessment of the actual locomotion performance in such difficult terrain. This leads to major inaccuracies, resulting in the infamous “reality gap”^25^.

To perform more effectively and intelligently, today’s legged robots must evolve^26^. Motivated by the imperative need to advance legged robot technology, this review paper addresses the challenges hindering their integration into diverse and unpredictable environments. These challenges, encompassing variations in terrains, obstacle avoidance, and adaptability to unforeseen conditions, underscore the necessity for robust control mechanisms. The exploration into dynamics, energetics, and system characteristics aims to bridge the current capability gap and envision a future where legged robots navigate any surface seamlessly.

Du et al.^27^ investigated a crawling mechanism that enables a soft robot to perform bidirectional locomotion using body deformation. The authors propose two different differential friction forces integrated into the robot’s body structure, allowing the robot to generate two different motion directions as the body deforms. With the proposed dynamic gaits, the robot can move in multiple directions with a simple system configuration and a minimalist actuation input. Carpentier et al.^28^ examined the dynamics and control of legged robots in terms of contact planning and trajectory optimisation. They gave an overview of the standard way to control legged robots, which is to set up a series of interactions with the environment. Kashiri et al.^29^ presented an overview of recent advances in the development of energy-efficient robotic systems with legs. They examined a number of robotic actuators that utilise compliance in parallel and series with the drivetrain to enable energy recycling for locomotion.

This study provides insight into the principles governing locomotion. It provides further details on models for the dynamics, energetics, and system characteristics. Mahapatra et al.^30^ evaluated the effects of walking parameters on energy consumption as well as static or dynamic robot stabilisation during gait generation on various surfaces. Meanwhile, Pardo et al.^31^ conducted a comprehensive literature analysis of scientific findings pertaining to legged locomotion on uneven terrain. Hongguang Li et al.^32^ this research proposes a vibration-driven robot powered by sinusoidal stiffness excitation, offering an alternative to force-based designs. A dynamic model and experiments using dielectric elastomer actuators show the robot can move in both directions by adjusting input frequency. The study reveals a new nonlinear mechanism and expands the application of smart materials in robotic locomotion.

To use robots in dangerous and unpredictable situations, especially on uneven terrain, they should walk more like humans and animals^33^. They are able to move on various surfaces resembling people or insects as biomimicry. The study of legged robotics, such as bipedal robots, informs the design and operation of assistive devices and applications. They also help study human and animal mobility. Robotic exoskeletons and prosthetic legs can give patients superhuman abilities like walking and running^34^. Numerous studies and reviews have been done, as this is such a promising area for research.

This study contributes to the advancement of vibration-driven locomotion by introducing a novel U-shaped elastic beam design that enhances stability and directional control. Unlike conventional vibration-driven robots that rely on elastic legs^2,14^ or bristle-based structures^6^, the U-beam structure provides an effective means of translating oscillatory forces into forward locomotion while reducing undesired lateral deviations. Furthermore, the proposed open-loop control strategy leverages the natural resonance of the beam, enabling efficient motion with minimal actuation requirements. This combination of structural innovation and control simplicity distinguishes the present work from prior approaches and underscores its potential for developing compact, low-cost, and energy-efficient vibration-driven robotic platforms approach validated through both simulation and experimental results.

This work aims to construct and evaluate a vibration-driven terrestrial robot utilizing a U-shaped elastic beam powered by a single DC motor, with the objective of examining the impact of angular velocity on stability, deviation, velocity, and hopping performance. Experimental studies at ω_1_ = 125.66 rad/s, ω_2_ = 251.33 rad/s, and ω_3_ = 376.99 rad/s demonstrated that augmenting angular velocity improved performance, with body velocity increasing from 93.79 mm/s at ω_1_ to 276.43 mm/s at ω_3_, and hopping distance rising from 401.60 mm to 420.07 mm. Simulations accurately reflected experimental hopping distances (error < 3 mm at ω_3_) but underestimated velocity by 50–60%. This work’s innovation consists on achieving stable locomotion by torsional vibration of a U-shaped elastic beam using a single actuator and open-loop control, therefore obviating the necessity for intricate feedback systems while preserving efficiency and stability.

## Modeling

**Fig. 1:**
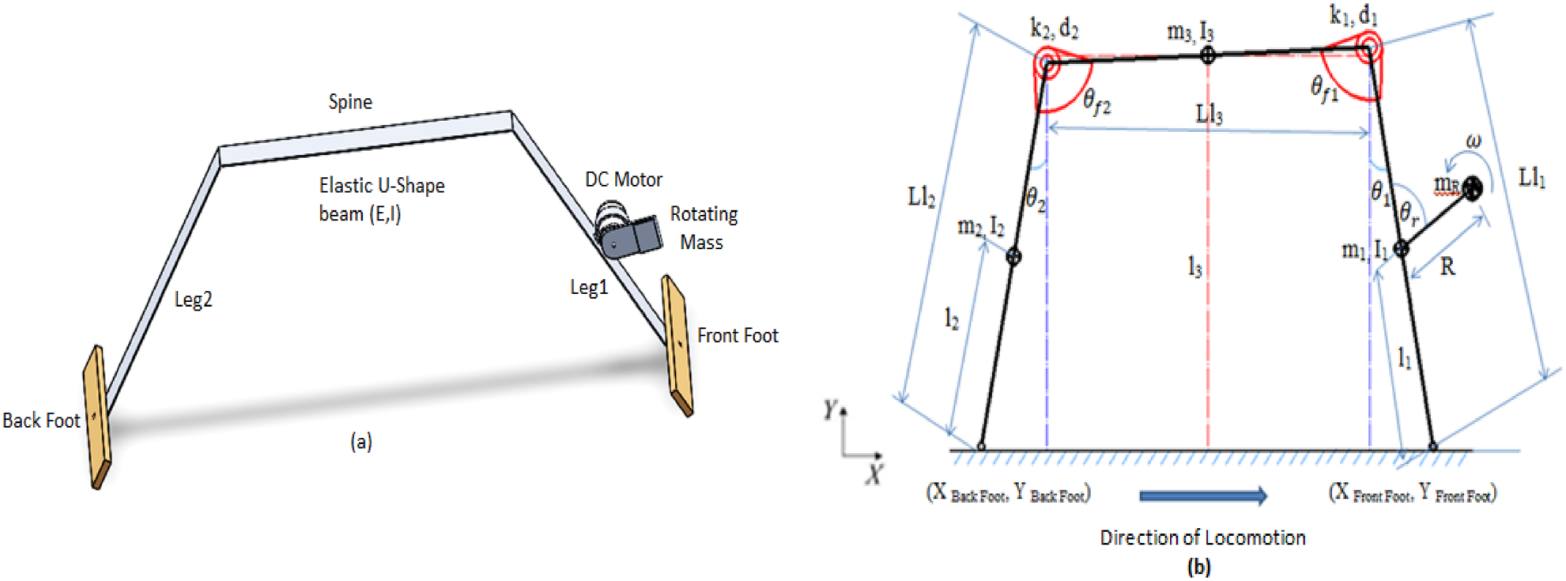
(**a**) Running robot made from U-shape elastic beam, (**b**) Parallel three link model of the robot.

The bounding mechanism introduced in this research features a U-shaped elastic beam driven by means of a pendulum see Fig. 1(a). The dynamics of such an elastic beam can be examined through discrete elements linked by spring-damper systems, with greater accuracy achieved as the number of these elements increases. Our approach demonstrates that the elastic beam can be effectively modelled using merely three links, illustrating planar quadruped movement refer to Fig. 1(b).

The following section describes the conceptual models of the mechanical structures and the motor control and defines some basic parameters to characterize the basic locomotion dynamics in this framework. The mechanism presented in this model consists of a DC motor, two large feet, and a main body made of a single-piece aluminium beam (E = 70 GPa) as shown in Fig. 1(a). The U-shaped beam constitutes the leg and spine of the robot, and a rotating mass is attached to the motor, which is mounted on the centre of the leg. An actuator controls the direction and the magnitude of the angular velocity of the rotating mass. We used two large feet to ensure that the motion of the robot was in the sagittal plane.

### Assumptions and variables

To construct a conceptual model for these robots and enable a systematic simulation analysis, several simplifying assumptions were made. First, the robot’s behavior is analyzed within the sagittal plane, treating the ground contact of each foot as a single point of contact. The robot’s spine is modeled as a rigid beam, and its mass is included in the analysis. The motor is represented as a concentrated point mass located at the center of the leg. Although the legs themselves are considered to have negligible mass, each foot is modeled as a point mass. The rotational interaction between each leg and the spine is represented by a torsional spring-damper system, as illustrated in Fig. 1(b). Additionally, the longitudinal deflection of the legs is modeled using a linear spring-damper element. Both the torsional and longitudinal stiffnesses are assumed to be linear and invariant with respect to the shape and deformation of the robot’s structural elements. Finally, the robot’s actuator is modeled as a semi-circular segment (semiscap) operating with a constant angular velocity (ω).

Based on these assumptions, the developed model comprises three rigid links interconnected through torsional and linear spring-damper elements. The direction of the robot’s movement is determined by the relative angular displacement between the links. When the rotation is clockwise such as θ₂ is greater than θ₁, the robot moves to the left. Conversely, when the rotation is counter-clockwise and θ₁ exceeds θ₂, the robot moves to the right. This behavior reflects the coordination between the leg segments and their interaction with the ground, as governed by the spring-damper dynamics within the model.

These assumptions were made to reduce the model’s complexity and enable analytical tractability within the Simscape framework. Since the mass of each wooden foot and aluminum leg is small compared to the motor and rotating eccentric mass, their inertial contribution to the overall system dynamics was considered negligible. Similarly, adopting linear torsional and longitudinal stiffness allowed us to approximate the elastic response of the U-shaped beam without introducing nonlinear parameters that are difficult to calibrate experimentally.

However, these simplifications directly affect the accuracy of horizontal velocity predictions. As observed in the results, the simulation consistently underestimates body velocity by approximately 50–60% compared to experiments. This discrepancy can be attributed to neglect the inertial effects of the legs and the nonlinear stiffness characteristics of the beam, both of which play a significant role in energy transfer and forward propulsion.

This model consists of 12 mechanical design parameters ($\:{L}_{l1},\:\:{L}_{l2}{,\:\:m}_{1},\:{\:m}_{2},\:{\:L}_{l3},{\:\theta\:}_{f},\:{\:k}_{\theta\:},{\:d}_{\theta\:},{\:m}_{h},\:\:{m}_{f},{\:m}_{M},\:\:$R), one control parameter (ω). Also, the running locomotion for this robot can be represented by 12 state variables (X_Backfoot_, Y_Backfoot_, X_Frontfoot_, Y_Frontfoot_, Y_Motor_ and θ_r_ and velocities of those) as shown in Fig. 1(b). X_Backfoot_, Y_Backfoot_, X_Frontfoot_ and Y_Frontfoot_ represent the coordinates of the foot of the robot. Y_Motor_ represents the Y-coordinate of motor, which is located at the center of the leg. θ_r_ is the angle of rotation of rotating mass with respect to X axis.

### Free vibration system

Torsional vibration in the U-shaped beam is induced by oscillating a rotating mass at a constant frequency, which is driven by a DC motor. This controlled oscillation generates periodic torque, causing the beam to undergo torsional motion in response to the motor’s continuous rotational input. This torsional vibration is significantly affected by the actuation frequency (ω) of rotating mass (m_R_). To understand the correlation between actuation frequency (ω) and resonance frequency (ω_nf_).

**Fig. 2:**
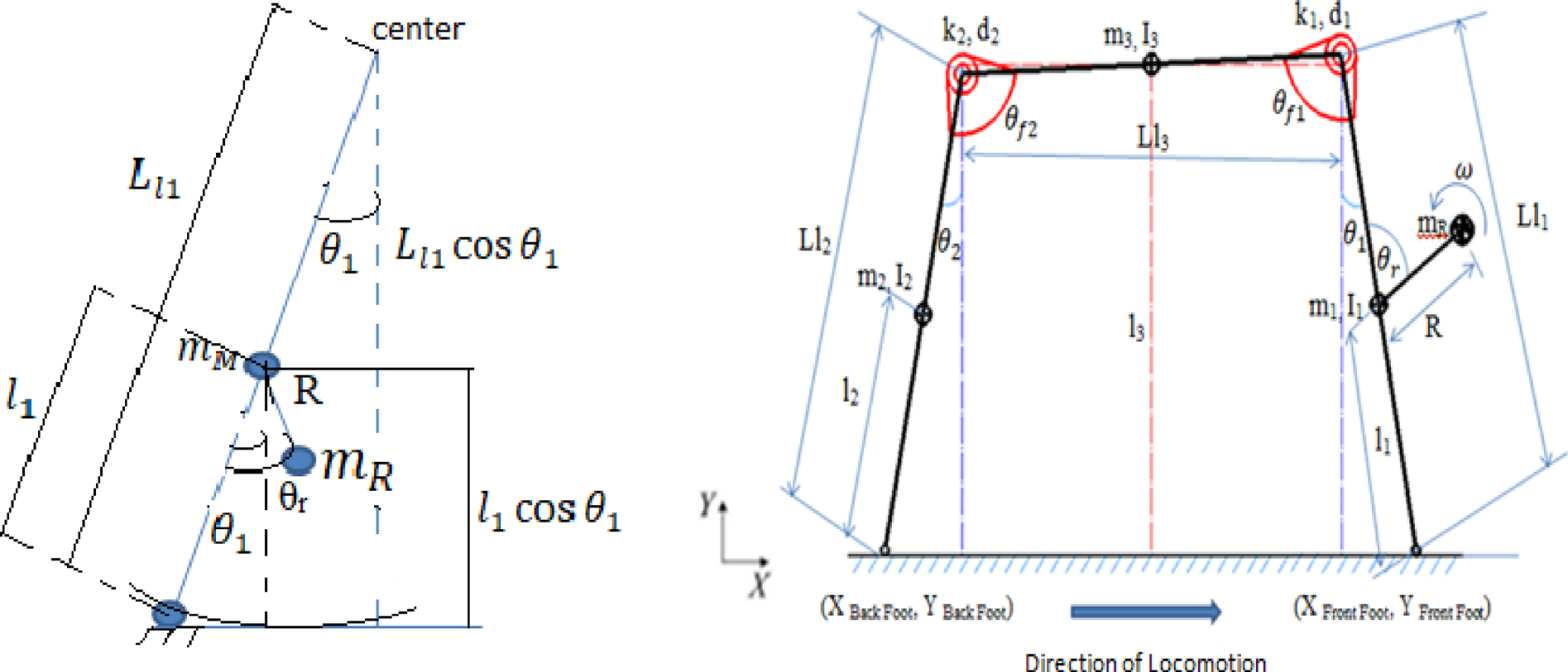
Schematic Diagram of the Torsional Locomotion Robot Model.

In this case, the Lagrangian $\:\mathcal{L}$ can be derived as:

1 $$\begin{array}{l}\mathcal{L}=\frac{1}{2}\left({m}_{total}\right){l}_{1}^{2}{\dot{\theta\:}}_{1}^{2}+\frac{1}{2}{I}_{1}{\dot{\theta\:}}_{1}^{2}+\frac{1}{2}{m}_{2}{l}_{2}^{2}{\dot{\theta\:}}_{2}^{2}+\frac{1}{2}{I}_{2}{\dot{\theta\:}}_{2}^{2}+\frac{1}{2}{m}_{3}{L}_{l1}^{2}{\dot{\theta\:}}_{1}^{2}\\ \quad\quad-\frac{1}{2}{m}_{3}{L}_{l1}^{2}{\dot{\theta\:}}_{2}^{2}+\frac{1}{2}{m}_{3}{y}^{2}{\dot{\theta\:}}_{1}^{2}-\frac{1}{2}{m}_{3}{y}^{2}{\dot{\theta\:}}_{2}^{2}+\frac{1}{2}{I}_{3}{\left(\dot{{\theta\:}_{1}}-{\dot{\theta\:}}_{2}\right)}^{2}\\ \quad\quad+\left({m}_{total}\right)g{l}_{1}\text{cos}{\theta\:}_{1}+{m}_{2}g{l}_{2}\text{cos}{\theta\:}_{2}-{m}_{R}gR+{m}_{3}g{(L}_{l1}+y)\text{cos}{\theta\:}_{1}\\\qquad+\frac{1}{2}{k}_{\theta\:1}{\left({\theta\:}_{1}-{\theta\:}_{f}\right)}^{2}+\frac{1}{2}{k}_{\theta\:2}{\left({\theta\:}_{2}-{\theta\:}_{f}\right)}^{2}\end{array}$$

By differentiating the Lagrangian ($\:\mathcal{L}$) with respect to torsion angle$\:\:{\theta\:}_{1}$, the torsional equation of the motion can be represented by:

2 $$\begin{gathered} \:\therefore \:\frac{{d\mathcal{L}}}{{d\theta {\:_1}}} = \left( {{m_{total}}} \right)l_1^2{\mathop {\theta \:}\limits^{..} _1} + {I_1}{\mathop {\theta \:}\limits^{..} _1} + {m_3}L_{l1}^2{\mathop {\theta \:}\limits^{..} _1} + {m_3}{y^2}{\mathop {\theta \:}\limits^{..} _1} \hfill \\\qquad\qquad\qquad\qquad\qquad\qquad - \left( {{m_{total}}} \right)g{l_1}{\text{sin}}\theta {\:_1} - {m_3}g({L_{l1}} + y){\text{sin}}\theta {\:_1} + {I_3}{\mathop {\theta \:}\limits^{..} _1} + {k_{\theta \:1}}\theta {\:_1} \hfill \\ \end{gathered}$$

For the small angles ($\:\text{sin}\theta\:\cong\:\theta\:)$ Eq. 2 can be linearized and torsional resonance frequency $\:{\omega\:}_{\theta\:1}$ of the U-shaped elastic beam can be obtained by using the homogeneous part of this differential equation:

3 $$\:{\ddot{\theta\:}}_{1}+{\omega\:}_{n}^{2}{\theta\:}_{1}=0$$

4 $$\:{\omega\:}_{n\theta\:1}=\sqrt{\frac{{k}_{\theta\:1}-\left({m}_{total}\right)g{l}_{1}-{m}_{3}g{(L}_{l1}+y)}{\left({m}_{total}\right){l}_{1}^{2}+{I}_{1}+{m}_{3}{L}_{l1}^{2}+{m}_{3}{y}^{2}+{I}_{3}}}$$

Where: $\:{k}_{\theta\:}$ represents the torsional stiffness of the substitute inter-segmental joints between the spine and the legs as shown in Fig. 1(b).

The expression of$\:{\:k}_{\theta\:}$ for a cantilever beam is given by EI/L. However, $\:{k}_{\theta\:}$ was determined experimentally in this study, since the elasticity of the aluminium cold-formed beams was not precisely known.

When the Lagrangian ($\:\mathcal{L}$) in Eq. 1 is differentiated with respect to torsion angle$\:\:{\theta\:}_{2}$,

5 $$\:\therefore\:\frac{d\mathcal{L}}{{d\theta\:}_{2}}={m}_{2}{l}_{2}^{2}{\ddot{\theta\:}}_{2}+{I}_{2}{\ddot{\theta\:}}_{2}-{m}_{3}{L}_{l1}^{2}{\ddot{\theta\:}}_{2}-{m}_{3}{y}^{2}{\ddot{\theta\:}}_{2}-{m}_{2}g{l}_{2}\text{sin}{\theta\:}_{2}-{{I}_{3}{\ddot{\theta\:}}_{2}+k}_{\theta\:2}{\theta\:}_{2}$$

For the small angles ($\:\text{sin}\theta\:\cong\:\theta\:)$ Eq. 5 can be linearized and torsional resonance frequency $\:{\omega\:}_{\theta\:2}$ of the U-shaped elastic beam can be obtained by using the homogeneous part of this differential equation:

6 $$\:{\ddot{\theta\:}}_{2}+{\omega\:}_{n}^{2}{\theta\:}_{2}=0$$

7 $$\:{\omega\:}_{\theta\:2}=\sqrt{\frac{{k}_{\theta\:2}-{m}_{2}g{l}_{2}}{{m}_{2}{l}_{2}^{2}+{I}_{2}+{m}_{3}{(L}_{l1}^{2}+{y}^{2})-{I}_{3}}}$$

The expansion of the Lagrange’s equations leads to the equations of the free vibrations of the robot in matrix form can be written as:

8 $$\begin{gathered} \:\left[ {\begin{array}{*{20}{c}} {\left( {{m_{total}}} \right)l_1^2 + {I_1} + {m_3}L_{l1}^2 + {m_3}{y^2} + {I_3}}&{\:0} \\ {\:0}&{\:{m_2}l_2^2 + {I_2} + {m_3}(L_{l1}^2 + {y^2}) - {I_3}} \end{array}} \right]\left[ {\begin{array}{*{20}{c}} {\mathop {\theta {\:_1}}\limits^{..} } \\ {\:\mathop {\theta {\:_2}}\limits^{..} } \end{array}} \right] \hfill \\ + \left[ {\begin{array}{*{20}{c}} {{k_{\theta \:1}} - \left( {{m_{total}}} \right)g{l_1} - {m_3}g({L_{l1}} + y)}&{\:0} \\ {\:0}&{\:{k_{\theta \:2}} - {m_2}g{l_2}} \end{array}} \right]\left[ {\begin{array}{*{20}{c}} {\theta {\:_1}} \\ {\:\theta {\:_2}} \end{array}} \right] = \left[ {\begin{array}{*{20}{c}} {{m_R}R\omega {\:^2}} \\ {\:0} \end{array}} \right] \hfill \\ \end{gathered}$$

## Simulation and experimental setup

The robot consists of a U-shaped beam from [AL] with thickness 1 mm. Legs length$\:{\:(\text{L}}_{\text{l}1},\:{\text{L}}_{\text{l}2})$ with mass$\:\:({\text{m}}_{1},\:{\text{m}}_{2})$. Spine length $\:\left({\text{L}}_{\text{l}3}\right)$ with mass $\:\left({\text{m}}_{3}\right)$ of the robot are made of torsional spring with stiffness $\:{(\text{k}}_{{\uptheta\:}})$ and damping coefficient $\:{(\text{d}}_{{\uptheta\:}})$, (the spring stiffness of internal segmental joint is determine by rigidity of material, moment of inertia of the beam and curvature of beam) with a cross section of t = 1 mm × b = 25 mm and formed as a U-shaped beam with right angles. Two feet made from very light wood with mass$\:\:({\text{m}}_{\text{h}}=\:{\text{m}}_{\text{f}})$. The micro-DC motor with mass motor$\:\:({\text{m}}_{\text{M}}$) at the center of the leg with radius (R) is driven by the motor. For each angular velocity, a minimum of three trials were conducted to ensure statistical reliability. Motion data were extracted from high-speed video footage using reflective markers tracked at 120 fps. The tracking was performed using MATLAB’s computer vision tools, enabling precise determination of marker positions in each frame. The red object tracking is designed to detect and follow red-colored objects in video sequences. It operates by processing each frame individually, isolating the target object based on its color, and superimposing the resulting trajectory onto the original video. Outliers, defined as data points exceeding ± 2 standard deviations from the mean, were excluded following standard practice. This ensures reproducibility and transparency in the results.

The photograph of the amphibious robot is shown in Fig. 1 and its main dimensions and inertial properties are summarized in Table 1. These values were derived from experimental measurements conducted on the actual robot prototype, as well as from material property data for aluminium and standard robotic actuators. Specifically, the longitudinal stiffness of the curved beam was experimentally determined to be 200 N/m, and torsional stiffness was determined from the mechanical properties of the aluminium beam using standard formulas for cantilevered beams.

**Table 1:** Main dimensions and inertial Properties.

Parameters	Descriptions	Dimensions
$\:{L}_{l1},\:{L}_{l2}$	Length of leg	$\:{L}_{l1}=\:{L}_{l2\:}=250\text{m}\text{m}$
t, b	Aluminium beam cross-section	1 mm * 25 mm
$\:{L}_{1},\:{L}_{2}$	Length from feet to the centre of mass of leg	$\:{L}_{1}=0.5{L}_{l1\:},\:{\:{L}_{2}=0.5L}_{l2}$
$\:{m}_{1},\:{m}_{2}$	Mass of leg	$\:{\:m}_{1}=\:{m}_{2}\:=\:16.8\:\text{g}\text{r}\text{a}\text{m}$
$\:{L}_{l3}$	Spine length	$\:{L}_{l3}=\:250\text{m}\text{m}$
$\:{L}_{3}$	$\:\text{E}\text{q}\text{u}\text{a}\text{l}\:{L}_{l2}\:,\:{L}_{l2}\:\text{W}\text{h}\text{e}\text{n}\:{\theta\:}_{1}={\theta\:}_{2}$	$\:{L}_{3}={L}_{l1}\text{cos}{\theta\:}_{1}\:or\:{L}_{l2}\text{cos}{\theta\:}_{2}$
$\:{m}_{3}$	Mass of spine	$\:{m}_{3}\:=\:16.8\:\text{g}\text{r}\text{a}\text{m}$
$\:{k}_{L}$	Longitudinal stiffness of the curved beam	200 (N/m)
$\:{k}_{\theta\:1},\:{k}_{\theta\:2}$	Torsional spring stiffens at spine	4 (Nm/rad)
µ_stick_	Stiction friction coefficient,	0.6
µ_slide_	Sliding friction coefficient	0.5
$\:{\theta\:}_{f1},\:{\theta\:}_{f2}$	Angle between leg and spine	$\:{\theta\:}_{f1}=\:{\theta\:}_{f2}={\theta\:}_{f}=120^\circ\:$
$\:{\theta\:}_{1},{\theta\:}_{2}$	Angle between leg and final point of spine	$\:{\theta\:}_{1}\ne\:{\theta\:}_{2}$
$\:{d}_{\theta\:1},\:{d}_{\theta\:2}$	Coefficient of damping	0.01 (Nm, s/rad)
$\:{m}_{h},\:{m}_{f}$	Mass of foot	$\:{m}_{h}={m}_{f\:}=\:20\:\text{g}\text{r}\text{a}\text{m}$
$\:{L}_{fr},\:{L}_{fl}$	Right and Left foot length	$\:{L}_{fr}=\:{L}_{fl}=\:150\text{m}\text{m}$
$\:{m}_{M}$	Mass of motor	40 gram
R	Radius of rotation	50 mm
$\:{m}_{R}$	Rotating mass	0.025 kg

One of the main advantages of using vibration based running locomotion is the use of only one actuator to control the speed of locomotion. The actuator used was a DC motor with a high gear reduction (6400 RPM), which ensured that the angular velocity of the rotating mass was not affected by the robot dynamics. The centripetal force induced by rotating mass on the robot structure is shown in this equation:

9 $$\:F\left(t\right)=\:{m}_{R}\text{*}R\text{*}{\omega\:}^{2}$$

The experimental setup involved controlling the angular velocity (ω) by adjusting the voltage supplied to the DC motor, without incorporating any sensory feedback, to achieve stable locomotion of the robot. A 955 mm-long track was constructed for the robot’s movement, with a reflective marker affixed to its body, as illustrated in Fig. 2 the entire process was captured using a high-speed camera operating at 120 frames per second, allowing for detailed analysis of the robot’s dynamic motion. Infrared light was used to track the reflective marker and accurately measure the locomotion.

**Fig. 3:**
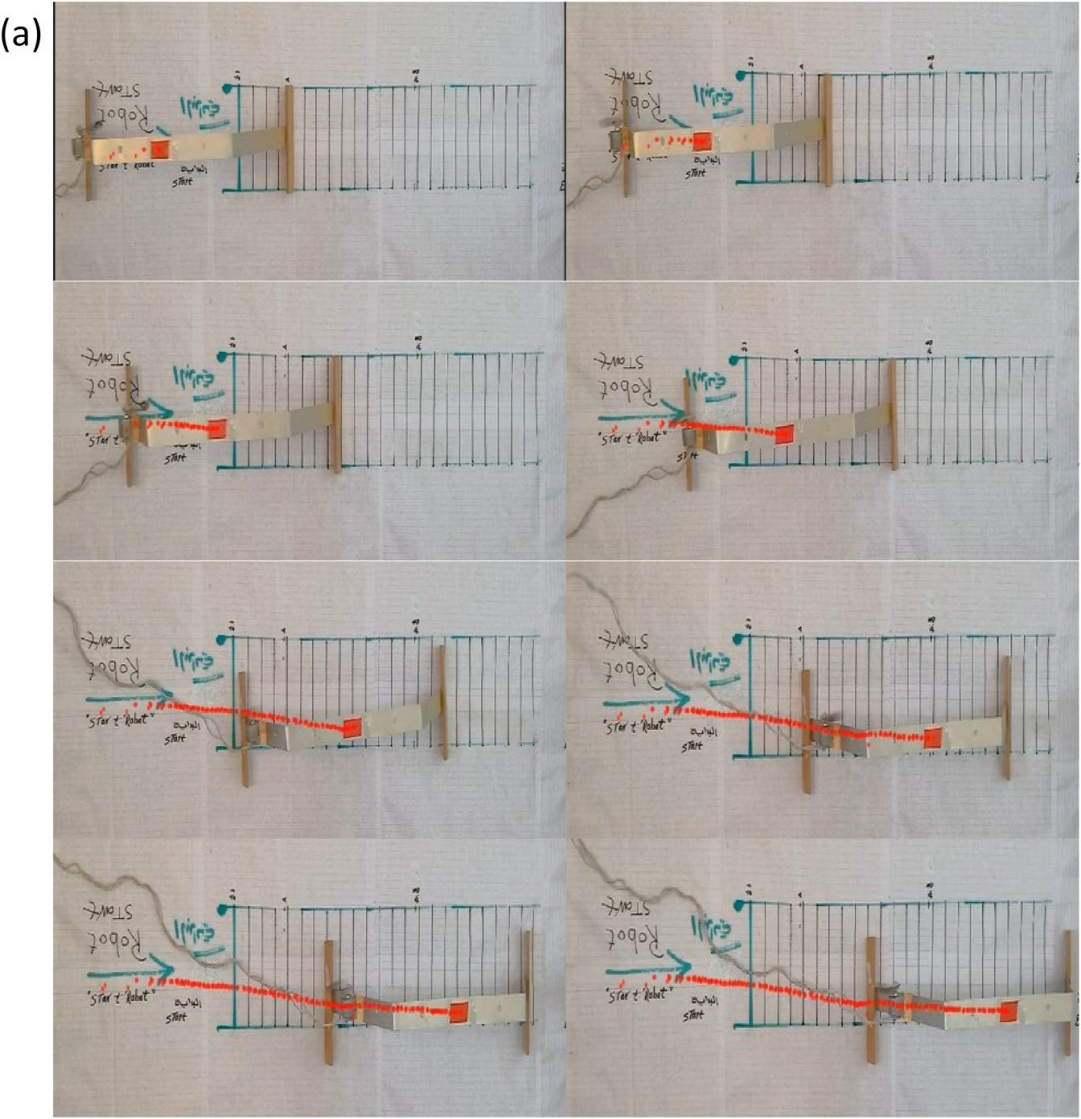

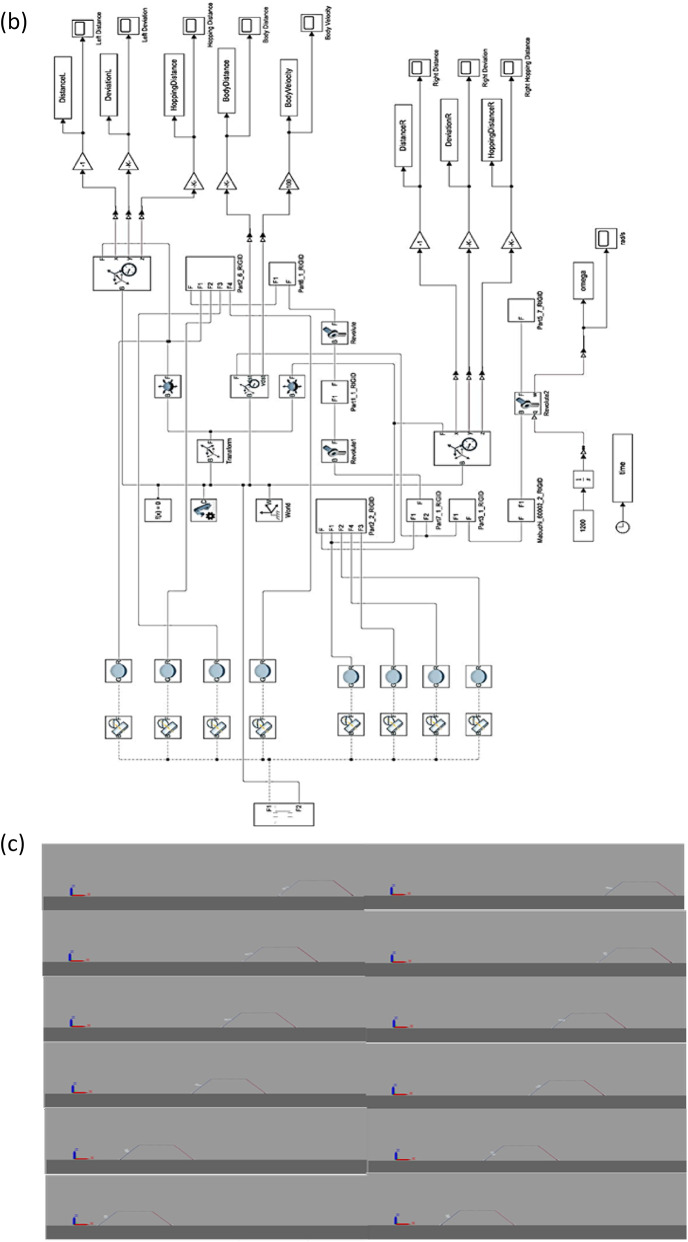
**a** Photographs of the model in real-world platform **b** Simulink Model of the Vibration-Based Locomotion Robot **c** Photographs for of the model simulation.

Figure 3.a presents a sequential photographic series illustrating the motion of the robot model on a real-world test platform. The images are arranged in pairs (left and right) across multiple rows to capture different moments in the robot’s locomotion cycle. The setup includes a clearly marked horizontal track, with uniform grid spacing used as a visual reference to quantify displacement and trajectory over time.

The surface of the platform has been marked with a baseline labeled “Robot Start” and additional reference lines for measuring position. The robot’s body is clearly visible, and a red reflective marker has been affixed near its center of mass to aid in motion tracking. The orange-red path traced by the marker across the successive images captures the trajectory of the robot as it progresses forward due to the torsional vibration mechanism driven by the internal rotating mass.

From the initial frame (top left), the robot is positioned at its starting location, and as we move through each pair of images downward, its position shifts progressively to the right along the track. The trajectory traced by the marker reveals a slightly curved and oscillatory path, which is characteristic of the locomotion induced by the internal torsional dynamics. The offset in marker positions between frames confirms the presence of asymmetrical actuation and alternating leg motion, consistent with the theoretical model discussed previously.

Additionally, the grid provides a quantitative basis for evaluating stride length and speed. The deflection and rotation of the legs can also be visually inferred from the change in angular orientation of the robot’s legs relative to the vertical axis in each frame. This visualization serves as practical validation of the conceptual model by offering insight into the real-world performance of the robot and the efficiency of the proposed locomotion mechanism. Overall, this figure plays a critical role in bridging the gap between simulation and experimental validation by showing the physical realization of the model and tracking its dynamic behavior over a fixed distance.

Figure 3.b illustrates the MATLAB Simscape model of the vibration driven U-shaped elastic beam robot outlined in this study. This figure illustrates a dynamic system wherein the robot’s body, legs, and spine are represented as rigid components linked by revolute joints, while torsional and linear spring-damper elements account for the compliance of the elastic beam. The input at the bottom of the picture is characterized by the angular velocity ω exerted on the eccentric rotating mass powered by a DC motor. This input produces the periodic torsional vibrations that drive the robot’s movement. The “World” block establishes the ground reference frame, whereas stiff transformations are employed to link the robot’s structure to its environment.

The diagram’s center illustrates the mechanical subsystems: the primary spine (U-shaped beam), left and right legs, and their interconnections via revolute joints. These elements align with the three-link conceptual model presented in the manuscript, wherein each link signifies a segment of the U-shaped elastic beam together with its corresponding stiffness and damping characteristics. The revolute joints facilitate angular displacement, permitting the simulation of oscillatory leg movement and engagement with the ground surface.

On the right side of the picture, several measuring subsystems are employed to capture essential locomotion data. The outputs for the left leg are DistanceL, DeviationL, and Hopping DistanceL, whereas the right leg yields DistanceR, DeviationR, and Hopping DistanceR. Moreover, the comprehensive bodily performance is assessed by BodyDistance and BodyVelocity. The simulation outputs directly align with the experimental observations detailed in your publication, in which displacement, deviation, velocity, and hopping distance were monitored using high-speed cameras and reflective markers.

In the simulation model, the angular velocity of the rotating mass (θ̇r) is assumed to remain constant throughout locomotion and is considered independent of the robot’s dynamic response. To analyze the dynamic behaviour of the U-shaped elastic beam robot, a physical representation of the system was implemented in MATLAB using the Simscape toolbox. The robot’s body is modelled as a U-shaped elastic structure, treated as a continuous beam to accurately capture its deformation and vibrational characteristics.

For the simulation experiments presented in this study, specific constant voltage values were experimentally determined to correspond to optimal locomotion performance. These voltages were selected based on their ability to induce the most stable and repeatable bounding gait patterns observed in the robot’s motion. The identification of these values was essential for replicating consistent dynamic behaviour and validating the performance of the modelled locomotion system.

Figure 3.c presents a series of simulation snapshots illustrating the dynamic locomotion behaviour of the U-shaped elastic beam robot modelled in MATLAB using the Simscape toolbox. The sequence is arranged in two vertical columns, each showing six time-stepped frames that capture the progression of the robot’s movement along a flat surface.

Each frame clearly depicts the robot body with its characteristic U-shape geometry and thin leg structures. The robot interacts with the ground through its feet, while the centre body undergoes periodic motion driven by internal torsional excitation. The X-, Y-, and Z-axis indicators in each frame provide reference for spatial orientation, confirming that the motion occurs predominantly along the X-direction (horizontal locomotion) while maintaining a constant elevation on the Z-axis (no vertical lift).

From the initial frame in the top left, the robot begins in a stationary position. As the sequence progresses downward and to the right, the robot’s center of mass is observed to shift forward, indicating effective translation. This motion results from the coordinated oscillation between the rigid links and the internal actuator, which was set to rotate at a constant angular velocity in the simulation. The leg segments appear to bend slightly at contact points, reflecting the spring-damper interactions embedded in the model.

By the final frame in the bottom right, a clear net displacement of the robot has been achieved. The symmetry of the gait and regularity of motion demonstrate that the chosen model parameters produce a stable bounding locomotion pattern. The simulation confirms the robot’s ability to move unidirectional without any active feedback control, validating the assumptions made in the conceptual model regarding constant actuator velocity and linear elastic behaviour of the beam and joints.

This figure serves as a critical visual validation of the numerical model, show casing the feasibility of torsion-driven locomotion and supporting the physical experiments presented earlier. It effectively bridges the gap between theoretical assumptions and practical motion outcomes observed in both simulation and experimental platforms.

## Results and discussions

### Effect of angular velocity on stability and deviation performance

**Fig. 4:**
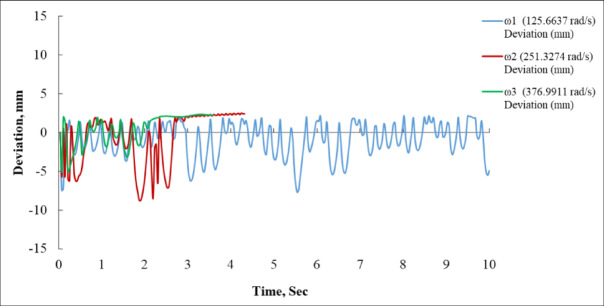
Deviation (mm) vs. Time (s) for Angular Velocities: 125.66, 251.33, and 376.99 rad/s.

Figure 4 depicts the temporal deviation (in mm) of a system under three distinct angular velocities: ω_1_ = 125.6637 rad/s, ω_2_ = 251.3274 rad/s and ω_3_ = 376.9911 rad/s. At the minimal angular velocity.

(ω_1_ = 125.6637 rad/s, depicted in blue), the system first undergoes minor oscillations around the zero line. After around three seconds, the deviation begins to swing more intensely, with magnitudes of up to ± 9 mm. The amplitude of these oscillations consistently rises throughout the period of the figure, indicating that the system is under-damped or exhibiting instability at this low velocity. The steady-state behaviour is ambiguous, and the deviation gets increasingly irregular, particularly after 5 s. This suggests that the system may be experiencing resonance or inadequate control feedback at this reduced input speed^16,35^.

Conversely, the system’s behaviour at a moderate angular velocity (ω_2_ = 251.3274 rad/s, depicted in red) demonstrates a more regulated reaction. The system initially experiences oscillations with significant amplitudes, reaching a minimum value of approximately − 9 mm at roughly 2.2 s. In contrast to the lower-speed scenario, the red curve indicates that the system starts to stabilize more rapidly^14^. Beginning at approximately 2.8 s, the oscillations diminish, and the deviation stabilizes at roughly + 5 mm. This indicates that the system is optimally calibrated at this velocity, with adequate damping to achieve a steady state following an initial transient phase. The deviance is consistently stable beyond 3 s, signifying that the system attains equilibrium under these circumstances.

The maximum angular velocity (ω_3_ = 376.9911 rad/s, indicated in green) produces the most stable and optimal system response. The curve initially exhibits rapid oscillations between ± 4 mm over the first 2 s; however, these oscillations possess reduced amplitude relative to the other two examples. At 2.5 s, the system attains a steady state with a deviation about + 3 mm and demonstrates negligible variations subsequently. The expedited stabilization and diminished overshoot indicate enhanced control at elevated angular velocities. The diminished oscillation amplitude and expedited settling time indicate that the system gains from higher-frequency input, either attributable to enhanced damping or decreased system lag.

When rotational velocity escalates, the system’s deviation behaviour markedly enhances. At reduced velocities, the system exhibits instability and demonstrates escalating oscillations over time. At moderate velocity, the system initially exhibits oscillatory activity but attains a stable steady state after around three seconds. At maximum velocity, the system achieves stabilization most rapidly and demonstrates the least overall variation. This signifies that the system’s efficacy is significantly influenced by the input angular velocity, with increased velocities yielding enhanced control, expedited settling, and less overshoot.

### Effect of rotational speed on distance coverage over time

**Fig. 5:**
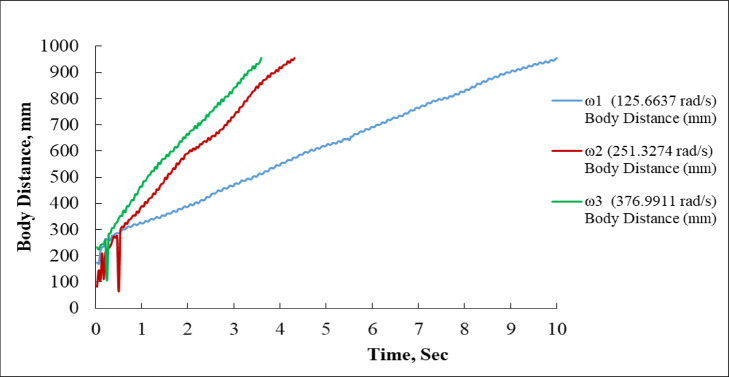
Body Distance vs. Time at Three Angular Velocities (ω_1_, ω_2_, ω_3_).

Figure 5 presents the variation of body distance (in mm) with respect to time (in seconds).At the minimal angular velocity (ω_1_ = 125.6637 rad/s), the system exhibits a consistent albeit diminished advancement in body distance. The process commences at approximately 200 mm and progressively ascends, to roughly 950 mm at the 10-second mark. The blue curve is smooth and uniform, signifying steady onward progression without sudden variations in pace. The travel rate is the slowest of the three speeds because of the reduced input angular velocity^36,37^.

The red curve, denoting ω_2_ = 251.3274 rad/s, initiates similarly near the 200 mm mark but rapidly escalates in gradient. From 0.5 s to 3.5 s, the body distance increases more steeply than ω_1_, attaining approximately 860 mm by 4.5 s, after which the curve ceases. This indicates that either the test concluded prematurely, or the system fulfilled its designated trajectory. The system operates at an accelerated pace, exhibiting minor oscillations; yet, the trajectory continues constantly upward with no indications of slowdown.

At the maximum angular velocity, ω_3_ = 376.9911 rad/s (green curve), the system exhibits the most rapid motion. Following a brief initial variation of 0 to 0.5 s, the distance between bodies increases swiftly. At 1 s, it crosses 400 mm, and at 3.5 s, it attains around 930 mm, beyond the red curve of ω_2_. Similar to the red curve, the green plot concludes about at the 4-second mark, indicating an earlier cessation of the test. The swift escalation in displacement verifies that the system’s body traverses the most distance in the least amount of time at this elevated rotational velocity.

A distinct link between angular velocity and body distance over time is seen when comparing the three charts. Increased angular velocity results in swifter and more extensive movement of the system within a specified duration. ω_3_ achieves the greatest displacement in the least amount of time, but ω_1_, albeit steady, has a significantly slower progression. These results demonstrate the direct impact of angular velocity on translational speed and efficiency in system movement^38^.

The graphic indicates that ω_3_ attains peak body displacement (≈ 930 mm) in merely 3.5 s, succeeded by ω_2_ (≈ 860 mm) within the same duration, whilst ω_1_ achieves a comparable displacement (~ 950 mm) only after 10 s. This behaviour is essential in applications requiring high-speed motion and reactivity, such as robots, actuator systems, or automated transport platforms^39,40^.

### Effect of linear velocity characteristics as a function of input angular speed

**Fig. 6:**
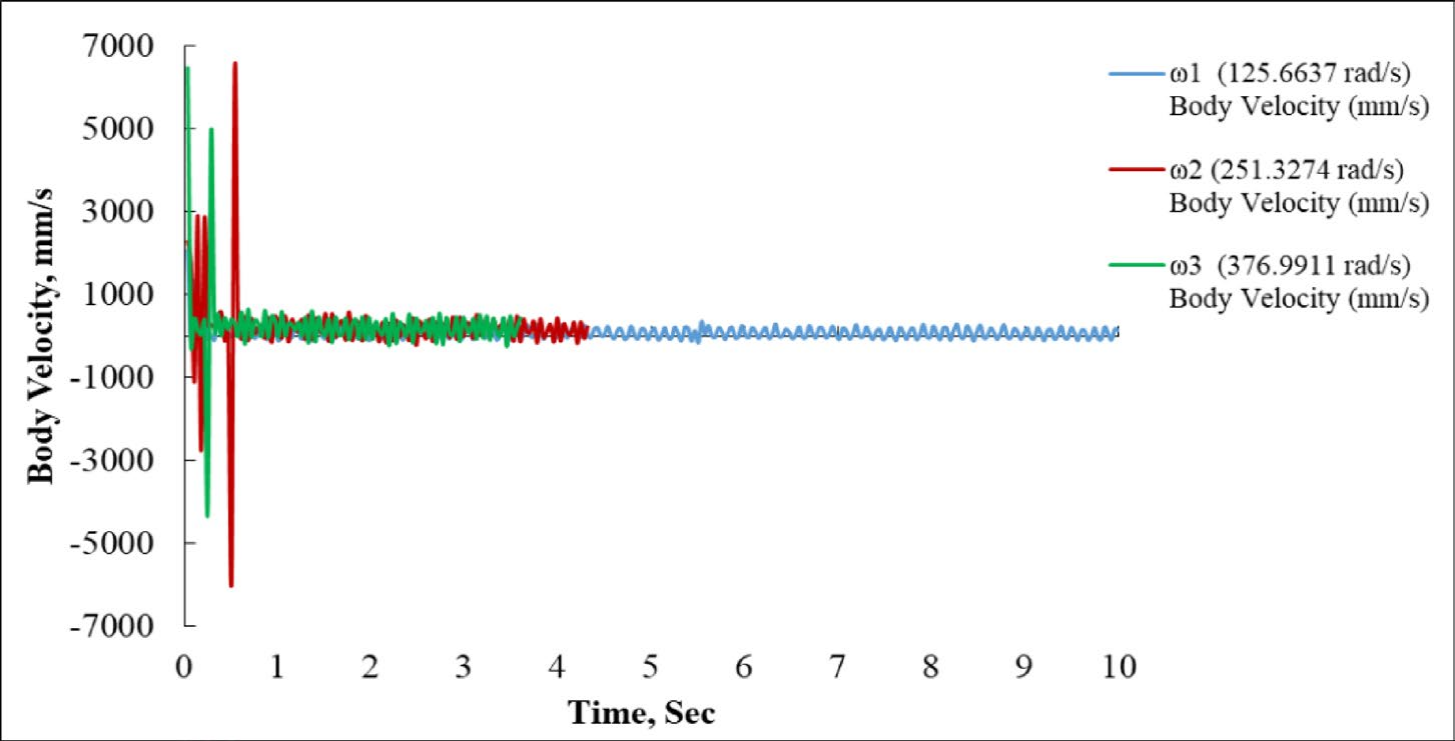
Body Linear Velocity vs. Time for ω_1_, ω_2_, and ω_3_.

Figure 6 illustrates the variation of body velocity in mm/s over a time range of 0 to 10 s, under three different angular velocity conditions.

At the lowest angular velocity (ω_1_ = 125.6637 rad/s), depicted by the blue curve, the system demonstrates a robust and consistent performance. Following a brief transient phase during the opening second, the velocity stabilizes within a limited oscillation range cantered at + 900 mm/s, maintaining this consistency until the 10-second interval. This curve has the steadiest performance of the three, characterized by minimum fluctuations and consistent oscillatory behaviour. The sustained velocity pattern for an extended duration signifies a well-damped and stable motion profile at this reduced input speed^35,36^.

Conversely, the ω_2_ = 251.3274 rad/s (red curve) and ω_3_ = 376.9911 rad/s (green curve) scenarios exhibit significant velocity changes, particularly during the initial transient reaction from 0 to 1.5 s. The red graph exhibits significant spikes, attaining about + 6000 mm/s and − 6000 mm/s, signifying strong acceleration and deceleration occurrences. Following this tumultuous onset, the red curve stabilizes into a damped oscillation pattern cantered at + 1200 mm/s, ultimately dissipating entirely around 4 s, indicating that the test or motion was prematurely concluded for this input^16,41^.

The green curve (ω_3_ = 376.9911 rad/s) exhibits the most significant initial instability, with velocity peaks surpassing + 5000 mm/s and plummeting to −5000 mm/s within the initial 0.5 s. Nonetheless, it stabilizes more rapidly than the red curve, achieving a more uniform oscillation pattern around + 1300 mm/s before finishing shortly after 3.5 s. This behaviour indicates enhanced system responsiveness and accelerated energy intake at elevated angular velocities, however accompanied by increased instability during the first period.

The graph’s most prominent feature is the inverse correlation between angular velocity and the duration of steady motion. ω_1_ maintains a somewhat stable body velocity throughout the 10-second interval, but ω_2_ and ω_3_ demonstrate more abrupt, transient reactions, stabilizing rapidly but only for a brief duration before halting. This may suggest that the system attains a maximum permissible distance or cutoff condition at elevated angular velocities, ceasing further movement^42^.

In conclusion, ω_1_ produces the most stable and sustained linear velocity, around 900 mm/s, with negligible disturbance. ω_2_ and ω_3_, despite attaining elevated peak velocities (up to ± 6000 mm/s), exhibit substantial transitory instability, resulting in their motion concluding markedly sooner (4 s and 3.5 s, respectively). The results underscore the trade-off between responsiveness and stability, indicating that increased angular speeds facilitate quicker movement but also present difficulties in control and motion continuity^39,40^.

**Fig. 7:**
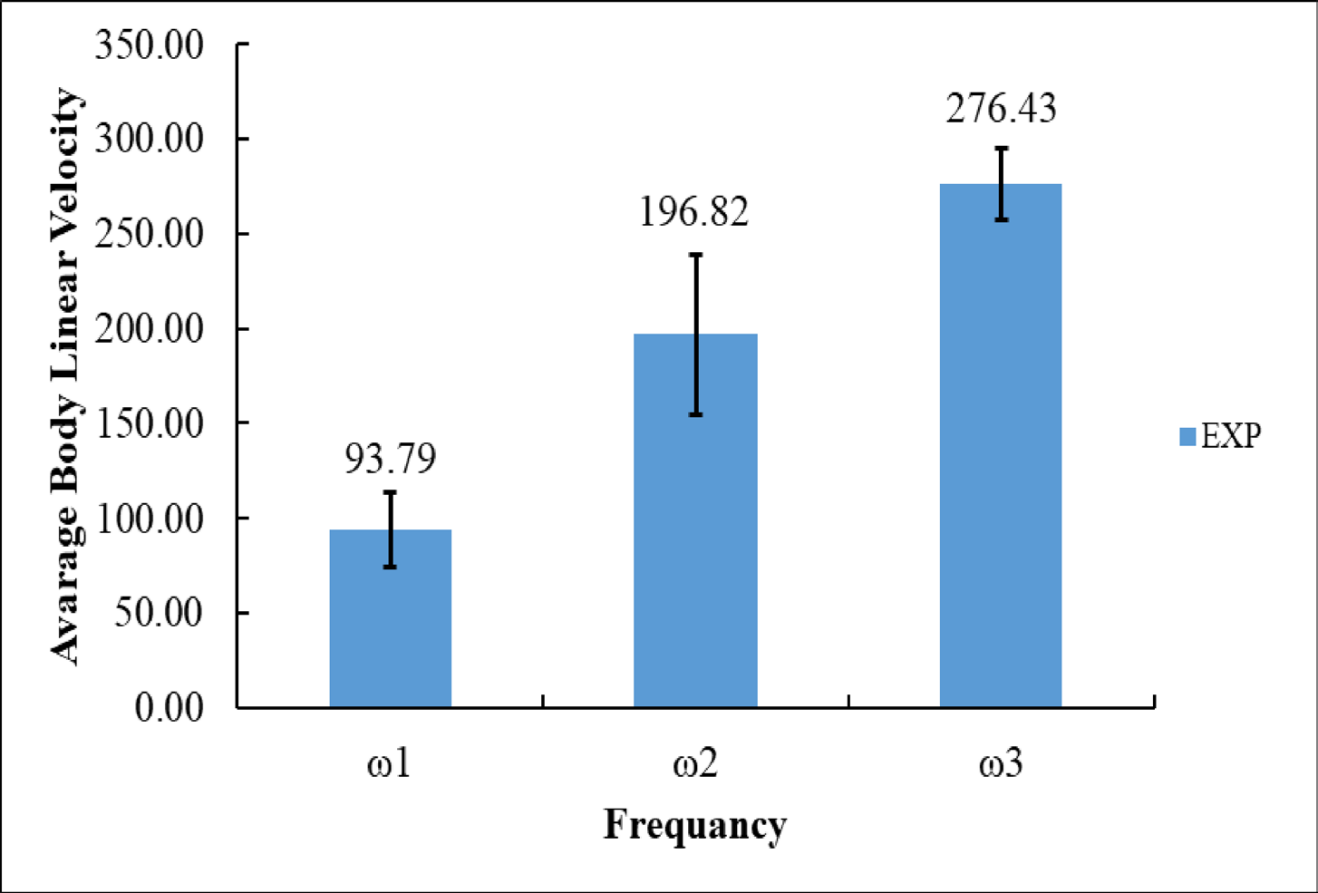
Experimental Average Body Linear Velocity at ω_1_, ω_2_, and ω_3_.

Figure 7 illustrates the experimentally recorded average body linear velocity (designated as EXP) at three specific angular frequencies, identified as ω_1_, ω_2_, and ω_3_.

At ω_1_, the minimum angular velocity evaluated, the system demonstrates an average linear velocity of 93.79 mm/s. This comparatively low value aligns with the system’s restricted movement at diminished input rotational speeds, as evidenced in the previous time-velocity charts^36^.

At ω_2_, a moderate angular velocity, the system exhibits a notable enhancement in performance, with the average linear body velocity increasing to 196.82 mm/s. This demonstrates that augmenting the rotational input more than doubles the average linear velocity relative to ω_1_, underscoring a robust positive link between angular speed and body velocity.

At the maximum angular velocity, ω_3_, the system attains optimal performance, achieving an average linear velocity of 276.43 mm/s. Despite the increment from ω_2_ to ω_3_ being less pronounced than that from ω_1_ to ω_2_, the rising trajectory remains significant and corroborates that elevated angular velocities yield accelerated linear displacement of the system.

### Effect of angular velocity on hopping performance and stability

**Fig. 8:**
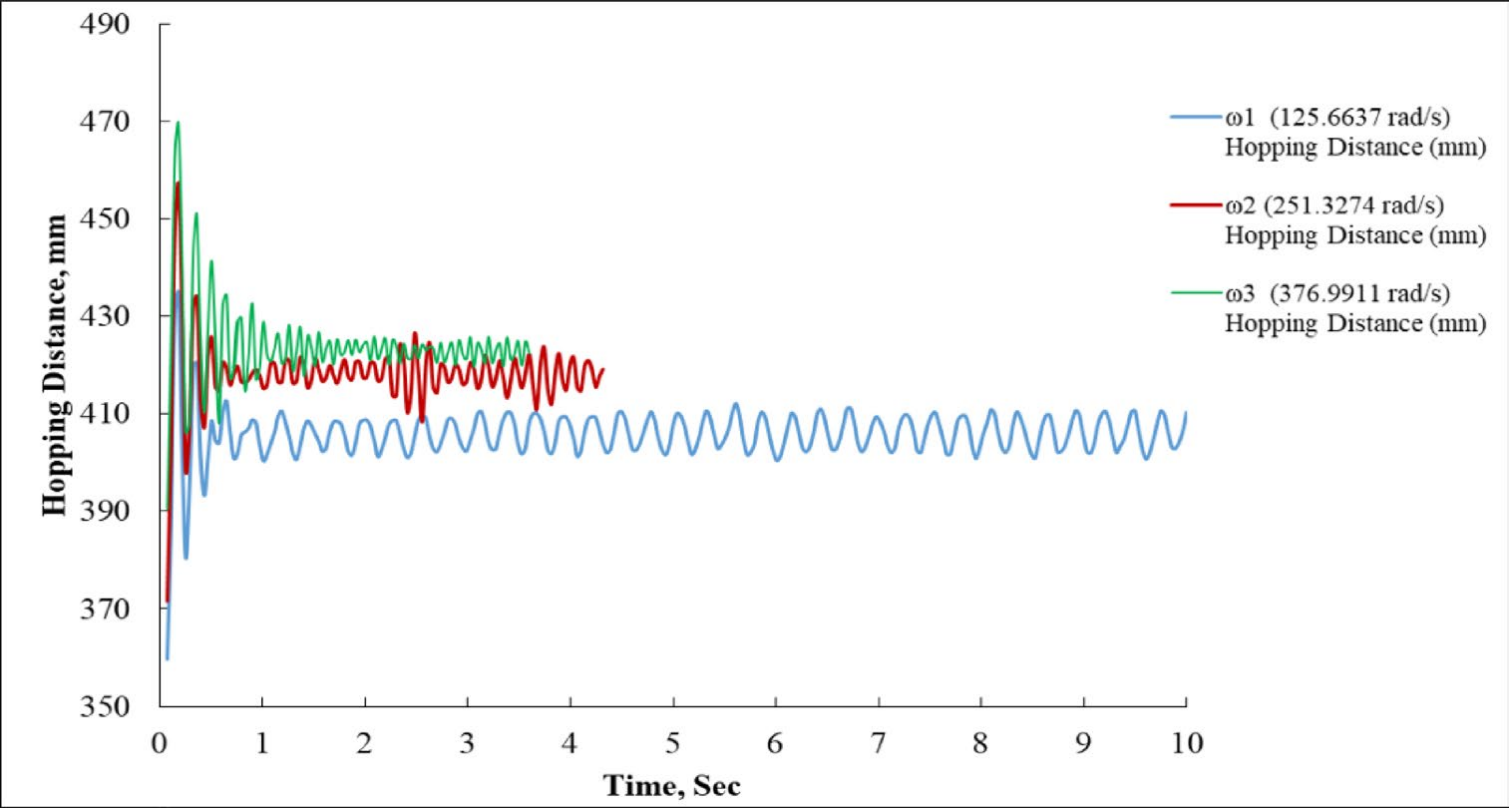
Experimental Measurement of Hopping Distance.

Figure 8 presents a time-series analysis of the vertical hopping distance (in mm) for a system operating at three different angular velocities.

At the lowest angular velocity, ω_1_ (blue curve), the system exhibits a smaller and consistent hopping amplitude. The oscillations start around 400 mm and settle into a steady oscillatory pattern with an average amplitude of around 10–15 mm. After the initial transient (within the first second), the hopping distance stabilizes near 405 mm, and the sinusoidal behavior continues with little variation throughout the full 10-second duration. This suggests that ω_1_ delivers stable but limited hopping power, resulting in modest but periodic jumps^36^.

For the mid-range angular velocity, ω_2_ (red curve), the hopping distance begins near 460 mm, and after an initial overshoot and quick damping within the first second, the curve settles close to 415 mm. The oscillations are smaller in amplitude than those of ω_1_ and ω_3_, indicating tighter control. The system maintains a relatively flat trajectory from 1.5 to 4 s, after which data for ω_2_ appears to stop, likely due to a limited test period. This result reflects a balanced hopping performance higher than ω_1_ but not the maximum observed.

At the highest angular velocity, ω_3_ (green curve), the system starts with the largest initial hopping distance, peaking just above 470 mm within the first second. This high peak is followed by a noticeable damping trend, as the curve descends toward 425 mm. From approximately 1.5 s onward, the hopping distance gradually stabilizes around 420–425 mm before the data ends near 4 s. The curve suggests that ω_3_ provides the highest initial energy input, resulting in the largest hops, but also shows some control challenges during the settling phase due to greater oscillation amplitude early on^2^.

**Fig. 9:**
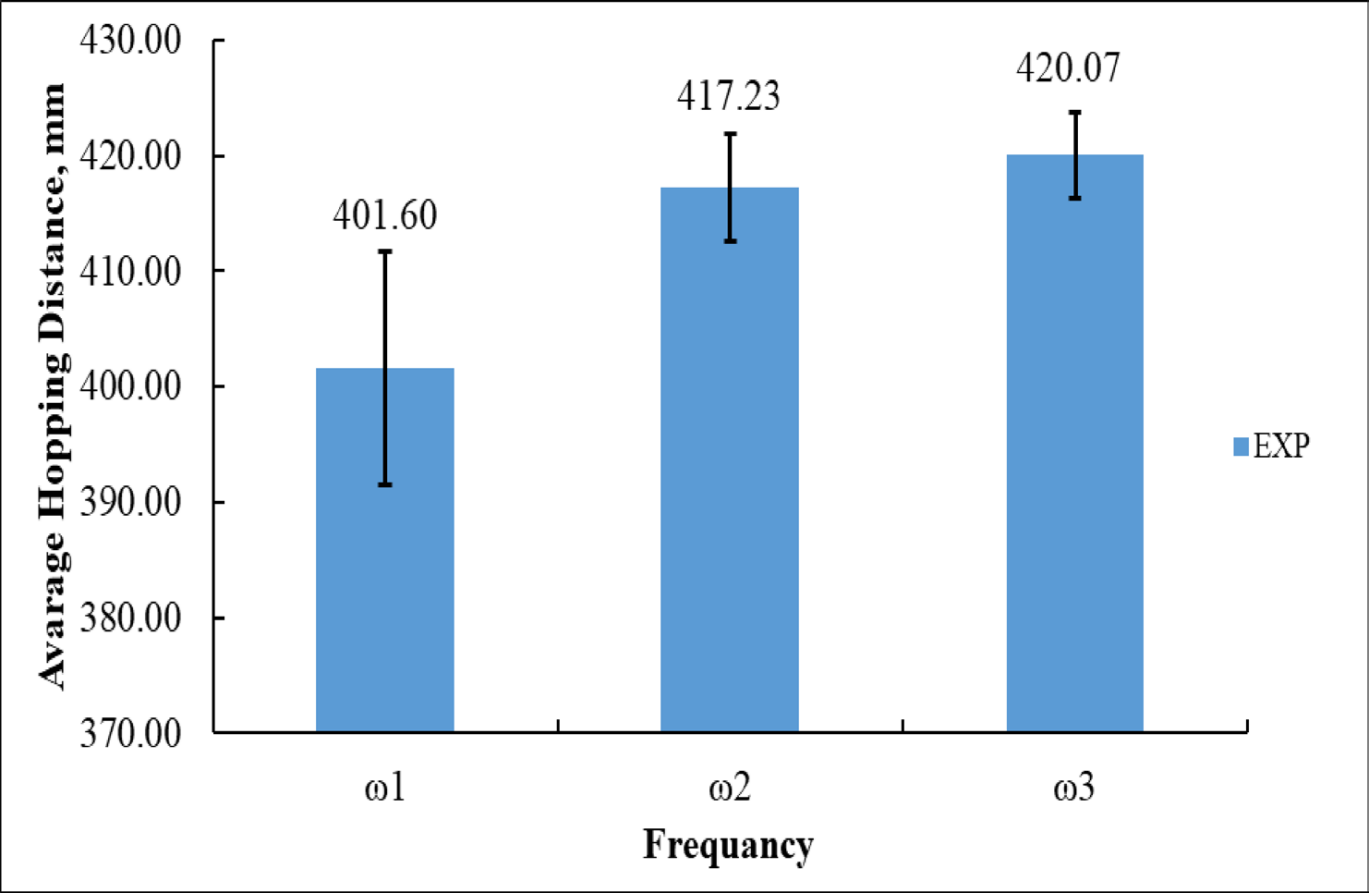
Experimental Average Hopping Distance at ω_1_, ω_2_, and ω_3_.

Figure 9 depicts the experimental outcomes of the mean hopping distance of the vibration-driven U-shaped beam robot at three distinct input angular velocities (ω₁, ω₂, and ω₃). At the minimal rotational velocity, ω₁ = 125.66 rad/s, the robot attained an average hopping distance of 401.60 mm, accompanied by a substantial error margin of approximately ± 10 mm, signifying diminished stability and less effective energy transfer at low excitation levels. Upon increasing the angular velocity to ω₂ = 251.33 rad/s, the average hopping distance enhanced to 417.23 mm, accompanied by a reduced error margin (about ± 5 mm), indicating a more stable and consistent performance. At the peak angular velocity, ω₃ = 376.99 rad/s, the robot achieved the greatest average hopping distance of 420.07 mm, accompanied by the smallest error margin (about ± 3 mm), underscoring the system’s improved efficiency and less variability at elevated speeds.

The transition from ω₁ to ω₂ yielded an enhancement of roughly 15.63 mm in hopping distance, although the advancement from ω₂ to ω₃ was lesser, at around 2.84 mm. In comparing ω₁ and ω₃, the total enhancement was 18.47 mm, substantiating that elevated angular velocities facilitate more robust and stable hopping locomotion. The results quantitatively illustrate that the robot’s hopping ability dramatically enhances with angular velocity; nevertheless, the rate of improvement decreases at elevated speeds, suggesting that the system is nearing its performance limit.

The Table 2 presents a comparative analysis of three independent signal conditions or waveforms ω_1_, ω_2_, and ω_3_ defined by their temporal, spectral, spatial, and application-specific characteristics. The time range indicates that ω_1_ functions for the longest duration (up to 10 s), whilst ω_2_ and ω_3_ encompass shorter intervals of 4.5 and 4 s, respectively. This indicates that ω_1_’s signal is intended for operations necessitating prolonged activation or movement. The signal type categorizes ω_1_ as a square waveform, characteristic of binary or on/off control signals typically employed in actuation systems where discrete states prevail. Conversely, ω_2_ and ω_3_ are sinusoidal but differ in frequency and amplitude ω_2_ presents a high-frequency, low-amplitude sinusoidal signal ideal for precise, rapid oscillations, while ω_3_ features a lower-frequency, higher-amplitude sine wave suitable for examining slower, more pronounced system responses. The disparities are evident in the frequency attribute, with ω_2_’s elevated frequency being optimal for vibration testing and resonance identification, facilitating the analysis of system natural frequencies and dynamic behavior during rapid oscillations. ω_3_, characterized by moderate to low frequency, is more appropriate for damped harmonic analysis, emphasizing the attenuation of oscillations over time owing to system damping. The hopping range data, covering millimeter-scale displacements, transitions from ω_1_ to ω_3_, indicates an increase in motion amplitude from about 424.5 mm to 428.1 mm, which corresponds with variations in signal amplitude and desired physical displacements or sensor responses. The application suggestions succinctly illustrate the practical utility of each signal: ω_1_’s binary nature renders it suitable for actuation or incremental motion; ω_2_’s attributes correspond with vibration or resonance testing, where the detection of system response to high-frequency stimuli is essential, and ω_3_’s parameters enable the examination of energy dissipation and damping in oscillatory systems. The table presents a curated assortment of signals designed for particular experimental or control objectives, balancing time duration, waveform intricacy, spatial displacement, and frequency to enhance the analysis of mechanical or electromechanical systems across various dynamic behaviors.

**Table 2:** Summary of signal Types, Ranges, and recommended Uses.

Feature	ω_1_	ω_2_	ω_3_
Time Range	0–10 s	0–4.5 s	0–4 s
Signal Type	Square-like	Sinusoidal (high freq, small amp)	Sinusoidal (lower freq, larger amp)
Hopping Range (mm)	~ 424.5 to 426.2	~ 426.3 to 426.8	~ 427.4 to 428.1
Frequency	Moderate	High	Moderate to low
Application Suggestion	Binary motion/actuation	Vibration test/resonance	Damped harmonic analysis

### Analysis of simulated and experimental results at ω_1_

**Fig. 10:**
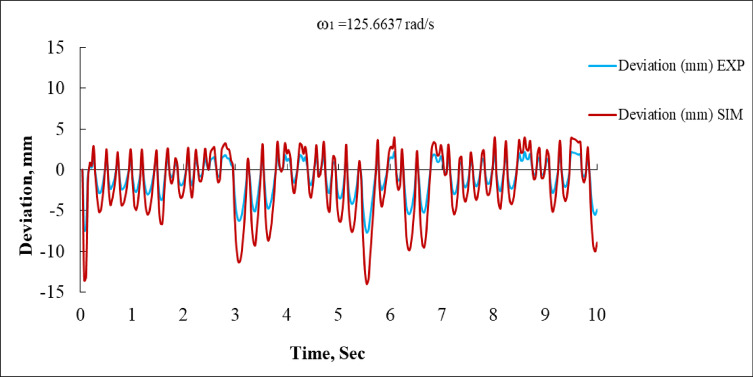
Deviation vs. Time at ω_1_ = 125.6637 rad/s (EXP vs. SIM).

Figure 10 demonstrates the deviation of the system in millimeters for both experimental (EXP) and simulated (SIM) outcomes over a 10-second interval. The blue curve (EXP) exhibits fluctuations predominantly within the ± 5 mm range, signifying relatively moderate oscillations. The red curve (SIM) exhibits oscillations of greater frequency and amplitude, with peak deviations of + 10 mm and − 14 mm occurring at t = 6.5 s. Although the simulated trend aligns with the experimental form in several instances, it exhibits overshoot, particularly in transient regions. The simulation appears to be more sensitive, either attributable to model assumptions or insufficient damping^16,35^.

**Fig. 11:**
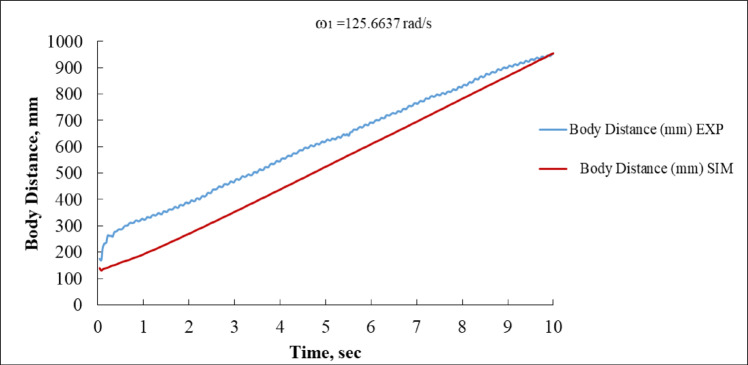
Body Distance vs. Time at ω_1_ = 125.6637 rad/s (EXP vs. SIM).

Figure 11 discusses the linear displacement (in mm) of the system’s body. The experimental curve (blue) initiates at approximately 200 mm and ascends sharply within the first second, attaining over 950 mm at 10 s. The simulation (red) exhibits a more linear development trajectory but underrepresents the displacement, commencing at around 120 mm and attaining over 900 mm at 10 s. A continuous distance disparity of approximately 30–50 mm exists between the EXP and SIM curves, indicating that the experimental system may have encountered increased momentum or reduced drag compared to the model^14,41^.

**Fig. 12:**
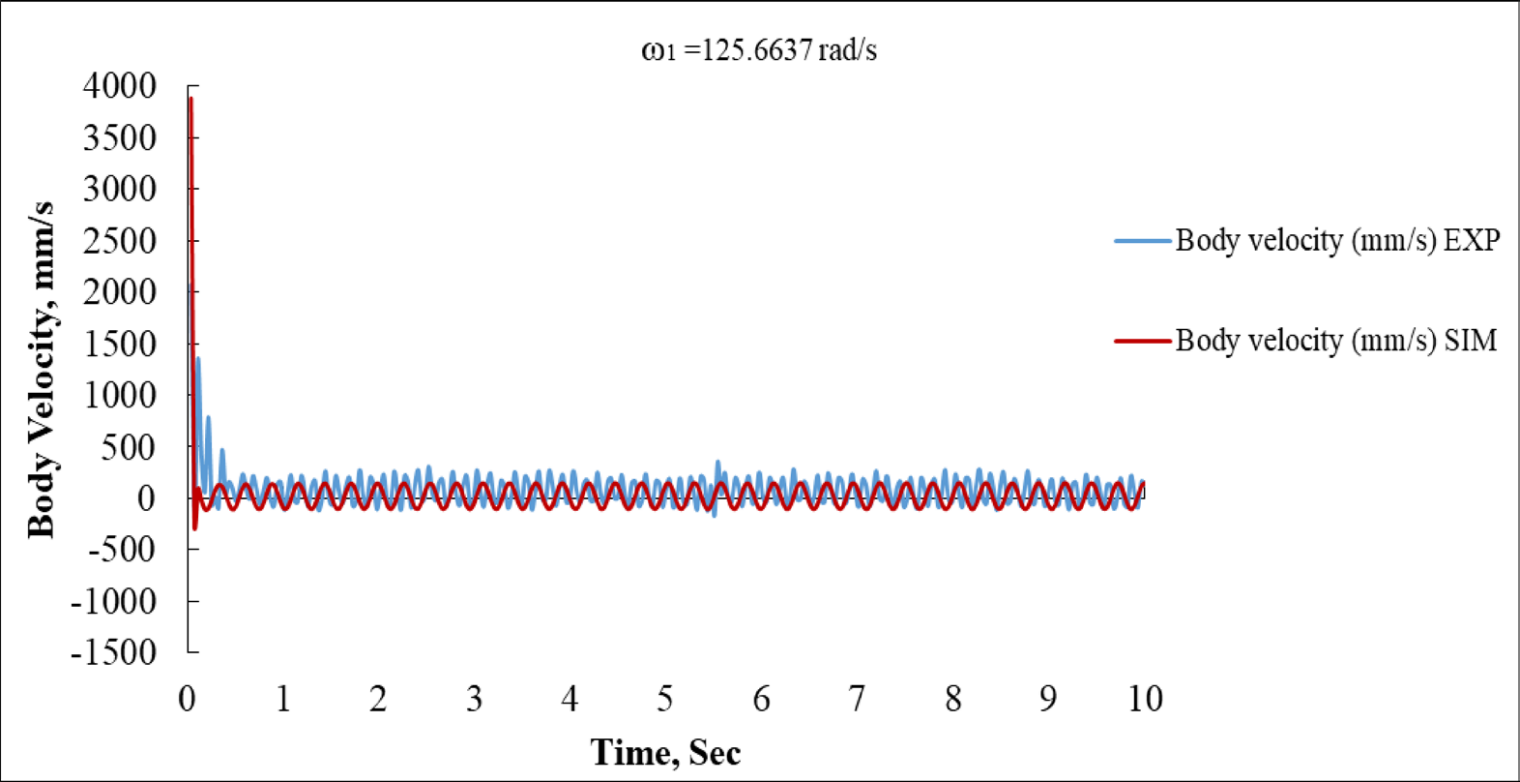
Body Linear Velocity vs. Time at ω_1_ = 125.6637 rad/s (EXP vs. SIM).

Figure 12 shows the body velocity in mm/s over time. The blue (EXP) curve shows a burst of energy within the first second, with a peak of about + 1500 mm/s, then settles into oscillations between ± 300 mm/s for the rest of the duration. The red (SIM) curve displays a sharp initial spike (nearly + 3700 mm/s) then quickly drops to oscillate within a similar ± 300 mm/s band. The initial spike in simulation is significantly larger than the experimental peak, which may be due to the model neglecting friction or inertia effects. After t = 1 s, both signals oscillate in a stable, nearly matched pattern, indicating good model fidelity during steady state.

**Fig. 13:**
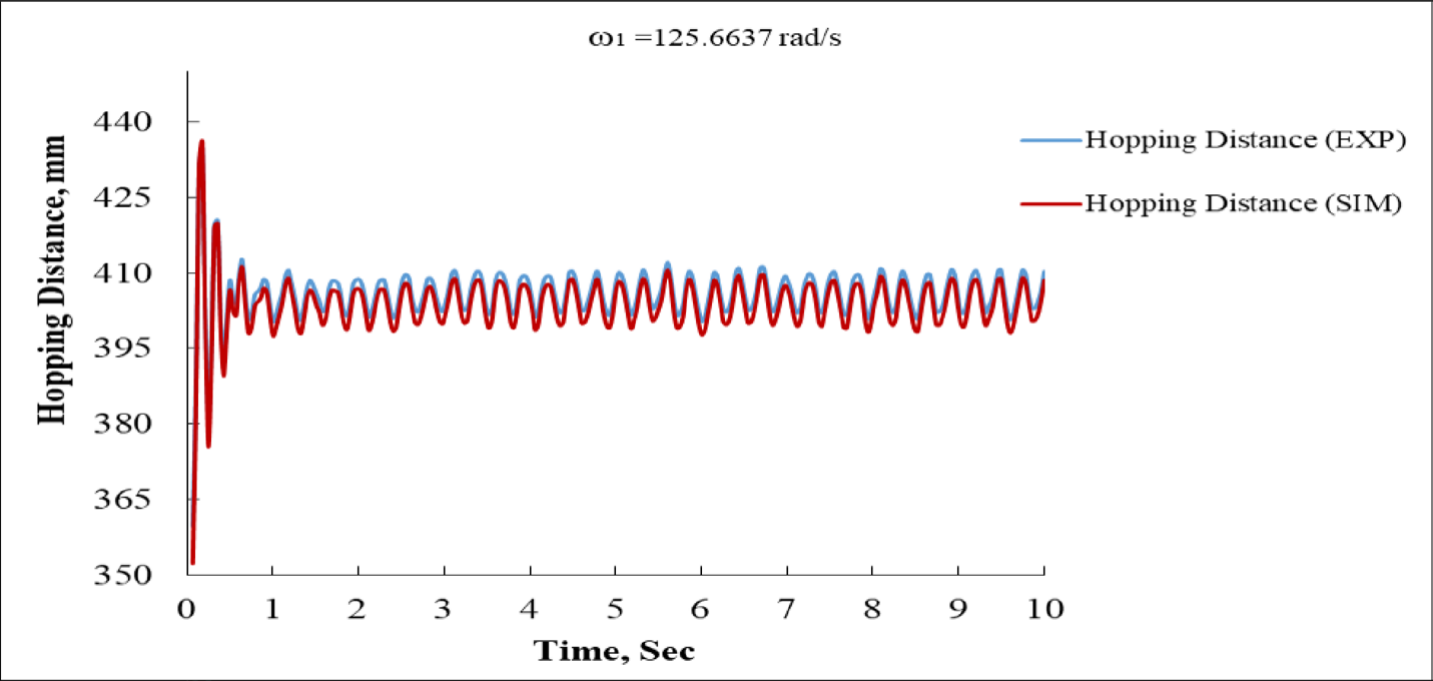
Hopping Distance vs. Time at ω_1_ = 125.6637 rad/s (EXP vs. SIM).

Figure 13 depicts the hopping distance (vertical jump height) of the system in mm. The blue curve (EXP) shows an initial overshoot near 430 mm, then stabilizes between 400 mm and 410 mm with consistent oscillations. The red curve (SIM) closely follows the experimental values after t = 1 s, stabilizing around 405 mm. The match between simulation and experiment is particularly strong in this figure, especially beyond the transient start up. The early mismatch could be attributed to differences in launch dynamics or actuator model tuning.

### **Analysis of simulated and experimental results at** ω_2_

**Fig. 14:**
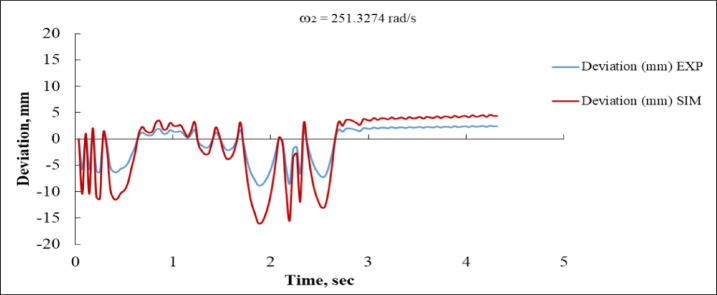
Deviation vs. Time at ω_2_ = 251.3274 rad/s (EXP vs. SIM).

Figure 14 depicts the temporal deviation for both experimental (EXP) and simulation (SIM) data at an angular velocity of 251.3274 rad/s. Initially, both curves demonstrate oscillating activity around the zero-deviation line; nevertheless, significant discrepancies emerge in their amplitude. The simulation curve attains a maximum variation of roughly − 20 mm at around 2 s, while the experimental curve stays within a somewhat narrower range of ± 7 mm. This mismatch indicates that the simulation model either overestimates the system’s sensitivity or is deficient in dampening^16,35^. After around 2.8 s, both responses stabilize around + 5 mm, demonstrating effective convergence in the steady state. The phase alignment is well preserved; however, the overshoot and amplitude discrepancies during the transient phase suggest potential enhancements for the simulation.

**Fig. 15:**
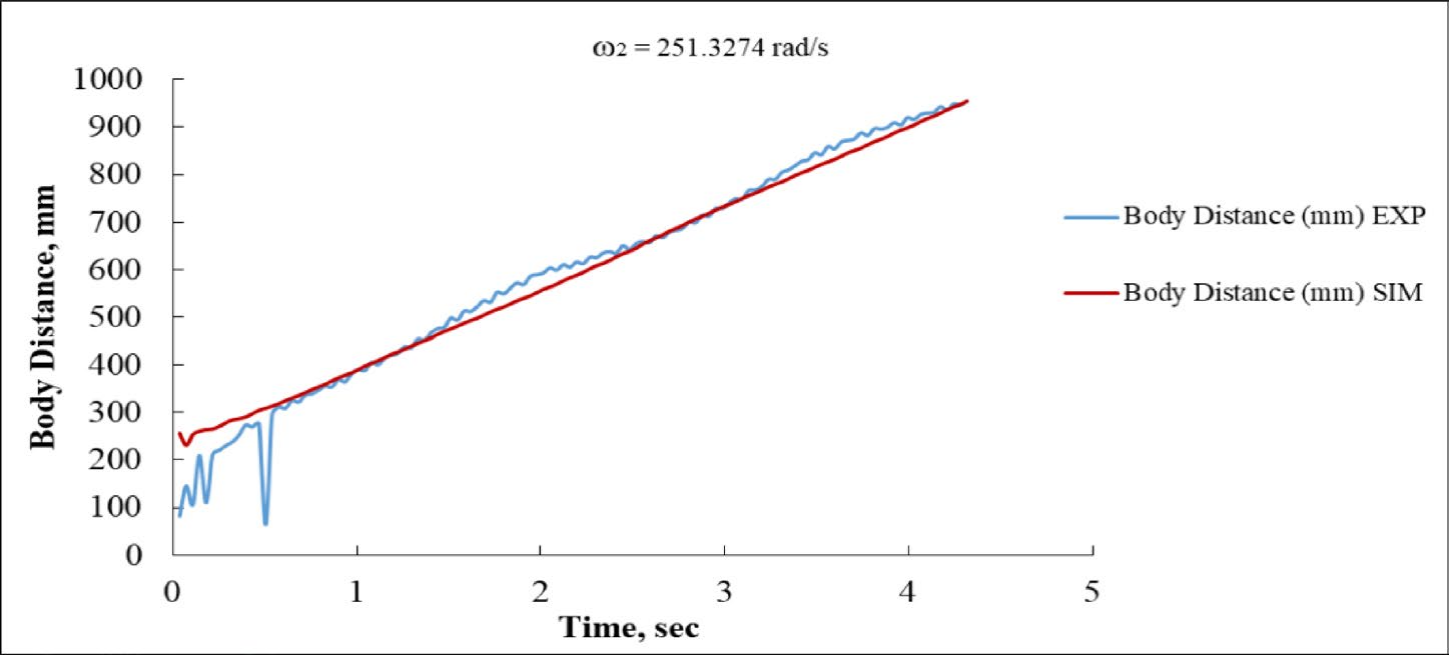
Body Distance vs. Time at ω_2_ = 251.3274 rad/s (EXP vs. SIM).

Figure 15 illustrates the relationship between body distance and time. The experimental response initiates with abrupt, unpredictable oscillations between 0 and 0.5 s, presumably attributable to mechanical instability or sensor noise. Notwithstanding this, it rapidly shifts into a linear growth phase. The body traverses a distance ranging from approximately 200 mm to exceeding 950 mm within duration of 5 s. In contrast, the simulated reaction initiates more gradually at approximately 150 mm, then adhering to a nearly linear path, consistently converging with the observed curve. Subsequent to the initial second, the simulation and experimental curves exhibit near-parallelism, maintaining a steady offset of roughly 30–40 mm, which signifies a satisfactory concordance in velocity trends, but with a minor underestimating in positional accuracy by the simulation model.

**Fig. 16:**
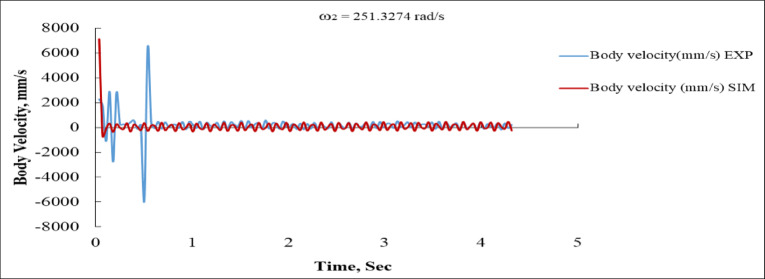
Body linear velocity vs. Time at ω_2_ = 251.3274 rad/s (EXP vs. SIM).

Figure 16 illustrates the body’s linear velocity as a function of time. The experimental curve first displays significant spikes, with values surpassing + 7000 mm/s and plummeting to almost − 7000 mm/s, indicating an unstable or abrupt commencement. This transient is prevalent in real-world systems characterized by mechanical compliance and feedback delay. The simulation curve initially spikes to around + 6500 mm/s, but subsequently stabilizes into steady oscillations. After one second, both curves stabilize inside a small band oscillating about ± 300 mm/s, indicating a significant correlation between experimental and simulated values in the steady-state domain^16,36^. The initial discrepancy underscores the difficulty of correctly replicating real-world start up conditions in a simulation.

**Fig. 17:**
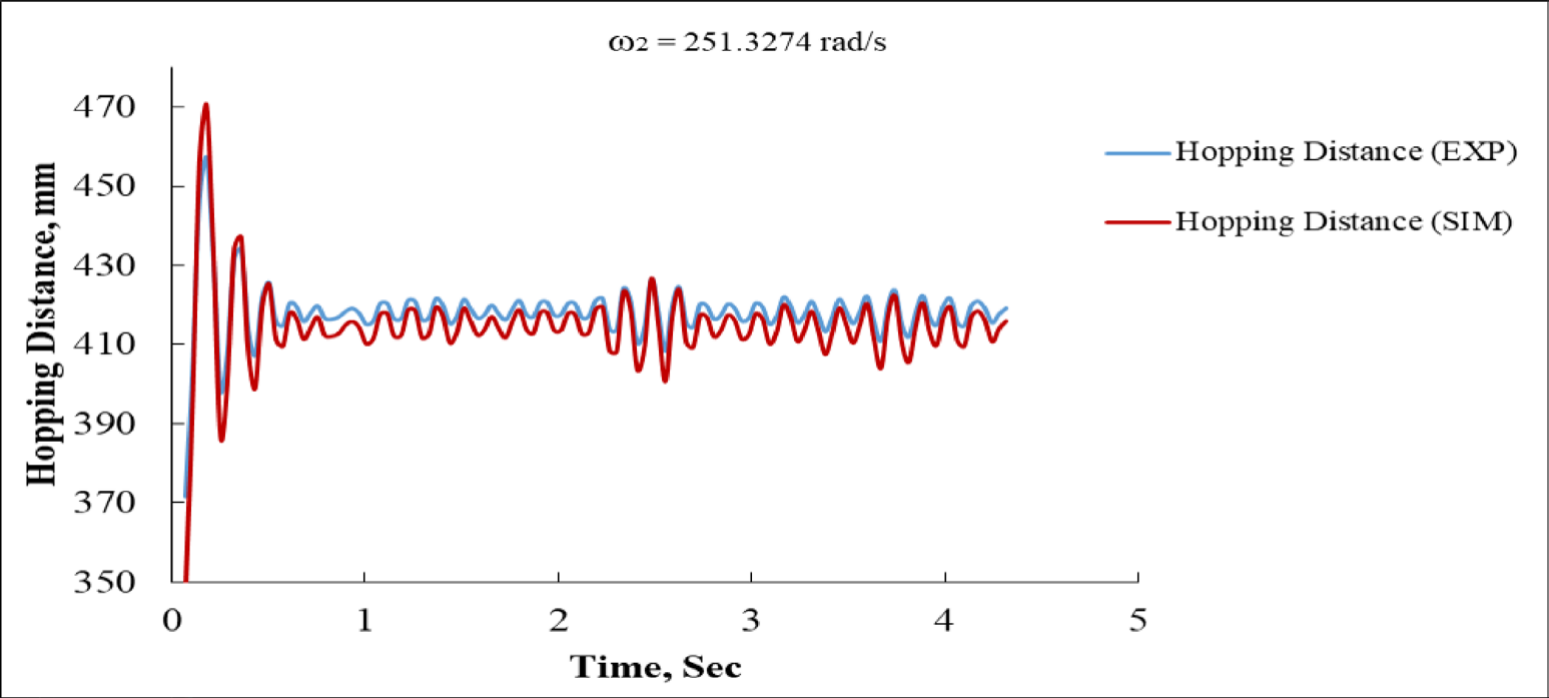
Hopping distance vs. time at ω_2_ = 251.3274 rad/s (EXP vs. SIM).

Figure 17 illustrates the hopping distance (vertical displacement) as a function of time for both the experimental and simulated systems. The simulation first attains a maximum of approximately 470 mm, then experiencing a steep decline below 350 mm, which is somewhat more pronounced than observed in the experimental data. Nonetheless, both curves attain stability rapidly. The experimental data fluctuates at 418 mm, whereas the simulation stabilizes at approximately 415 mm, exhibiting comparable amplitudes and frequencies. From roughly one second onward, the curves are closely aligned. This tight match indicates that the simulation model effectively captures the dynamics of hopping behavior at this angular velocity, especially during the steady-state phase.

### Analysis of simulated and experimental results at ω_3_

**Fig. 18:**
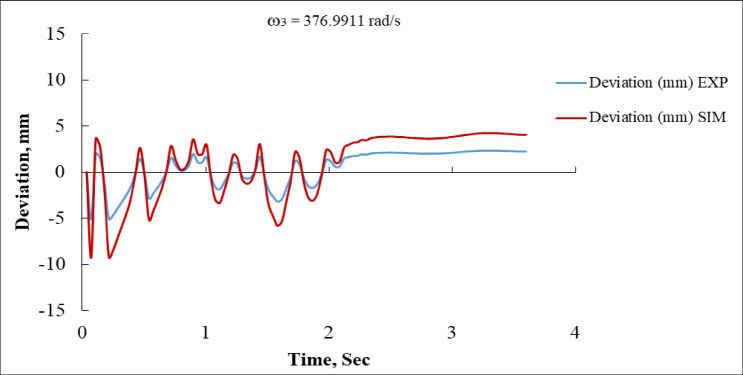
Deviation vs. Time at ω_3_ = 376.991 rad/s (EXP vs. SIM).

Figure 18 illustrates the deviation response over time for both the experimental and simulated results at an angular speed of 376.9911 rad/s. Initially, the deviation exhibits significant oscillations in both cases, with the experimental response reaching a maximum of approximately + 10 mm and a minimum of around − 8 mm, while the simulated response shows slightly larger excursions of up to + 12 mm and down to − 12 mm. These oscillations gradually decay as time progresses, with both responses settling within a range of ± 2 mm after roughly 2.5 s. The simulation, however, consistently demonstrates a slightly higher amplitude of oscillation and slower damping compared to the experimental results, suggesting that the simulation model may underestimate system damping or neglect certain dissipative effects present in the experimental setup^16,35^. Despite these differences, both curves ultimately converge to a stable value near zero deviation, indicating close alignment between the two approaches.

**Fig. 19:**
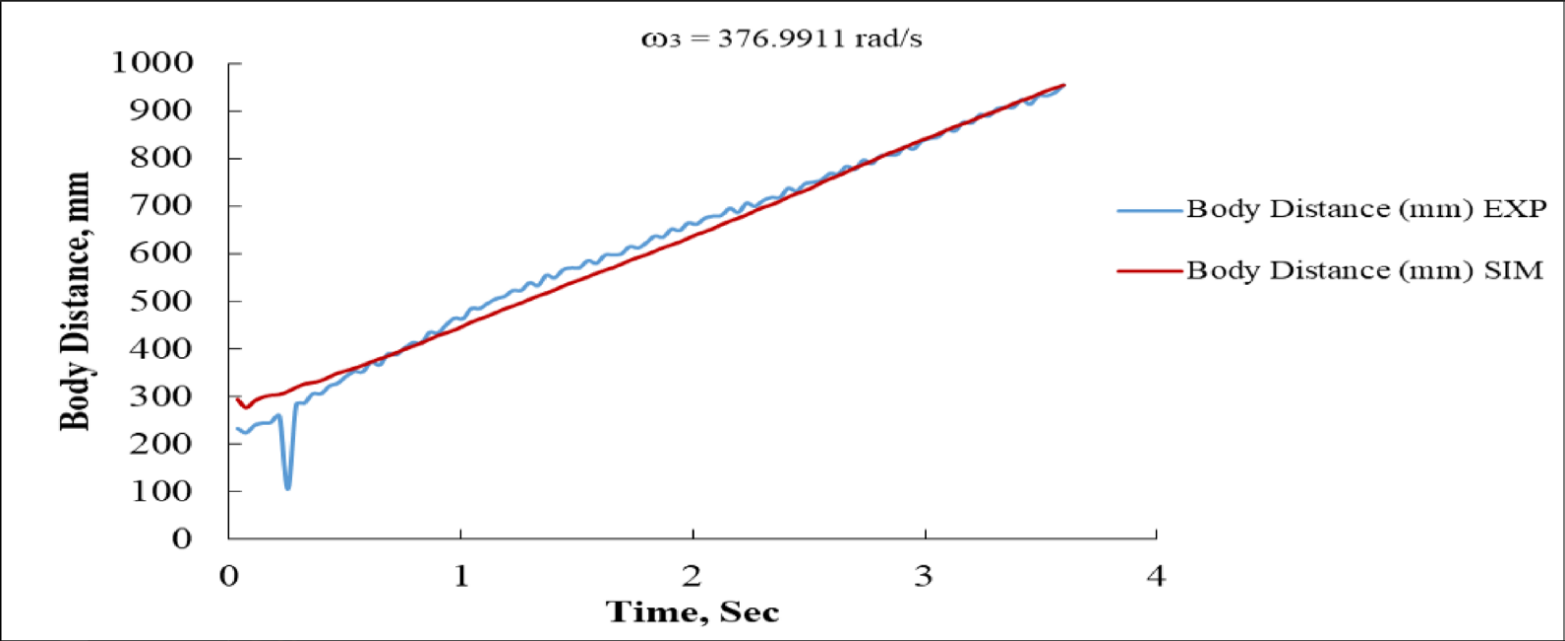
Body Distance vs. Time at ω_3_ = 376.9911 rad/s (EXP vs. SIM).

Figure 19, the body distance is plotted against time, reflecting the forward progression of the system. The experimental trajectory exhibits a nearly linear increase but with noticeable small fluctuations and an early dip at around 0.2 s, where the body distance briefly drops to nearly 250 mm before recovering. In comparison, the simulated data follow a smoother and more ideal linear progression, beginning at about 300 mm and increasing steadily. At 0.5 s, the experimental result records a displacement of about 320 mm compared to 310 mm for the simulation, while at 2 s the values are approximately 640 mm for the experiment and 620 mm for the simulation. By 4 s, both curves converge around 940–950 mm. This close agreement after the first second indicates that the simulation accurately predicts the long-term displacement trend, while the initial experimental irregularities may stem from physical disturbances such as surface slip, mechanical backlash, or measurement noise.

**Fig. 20:**
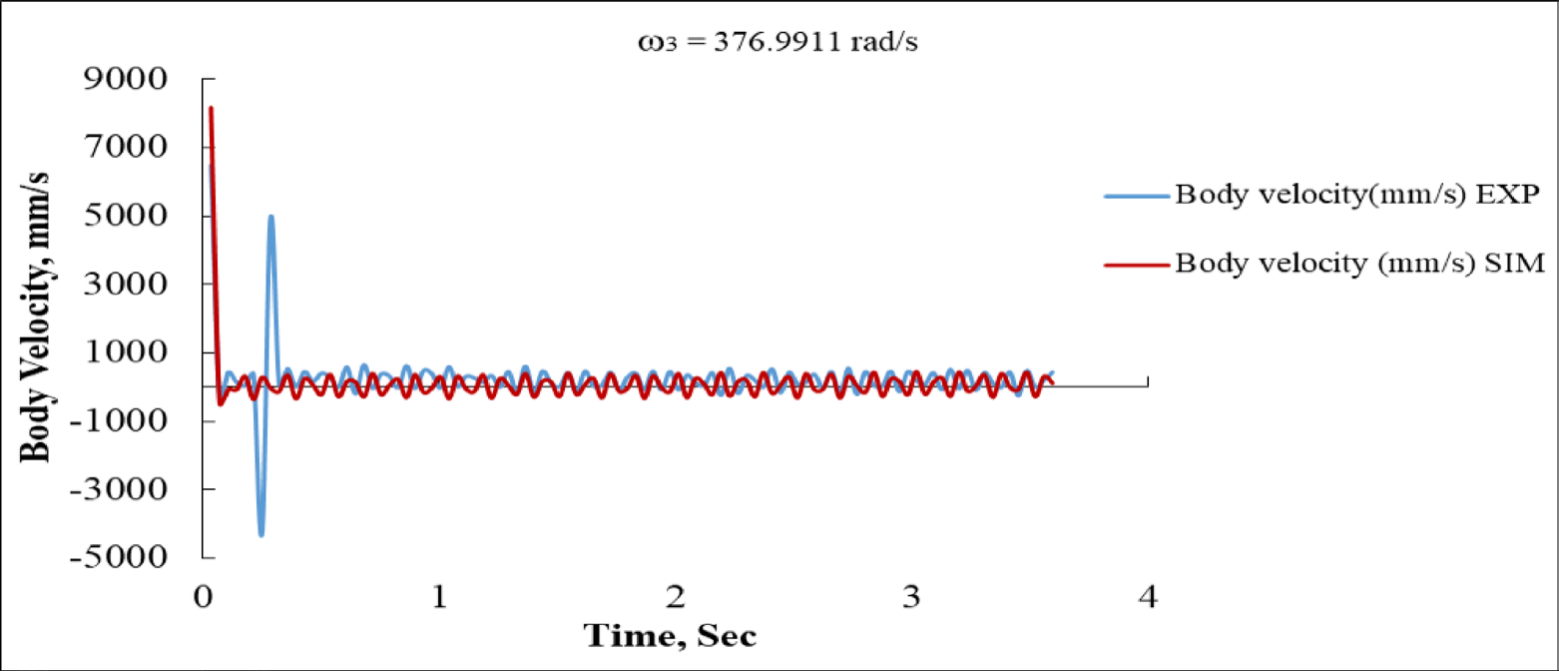
Body Linear Velocity vs. Time at ω_3_ = 376.9911 rad/s (EXP vs. SIM).

Figure 20 focuses on body velocity, where the system demonstrates a sharp transient phase before stabilizing into a quasi-steady oscillatory state. The experimental velocity response experiences a dramatic spike, peaking at approximately + 5000 mm/s within the first 0.1 s, followed shortly by a trough of nearly − 4500 mm/s. By contrast, the simulation predicts an even sharper initial spike, approaching + 8000 mm/s at the start, before settling more smoothly. After the first second, both responses stabilize with oscillations of smaller amplitude, fluctuating around ± 500 mm/s.

The discrepancy in the magnitude and smoothness of the initial velocity response indicates that the simulation assumes more instantaneous acceleration, while the experimental system is subject to inertia, frictional damping, and possibly delayed actuation^16,36^. Despite this, both experimental and simulated results exhibit similar long-term oscillatory patterns, demonstrating that the model successfully captures the essential steady-state dynamics.

**Fig. 21:**
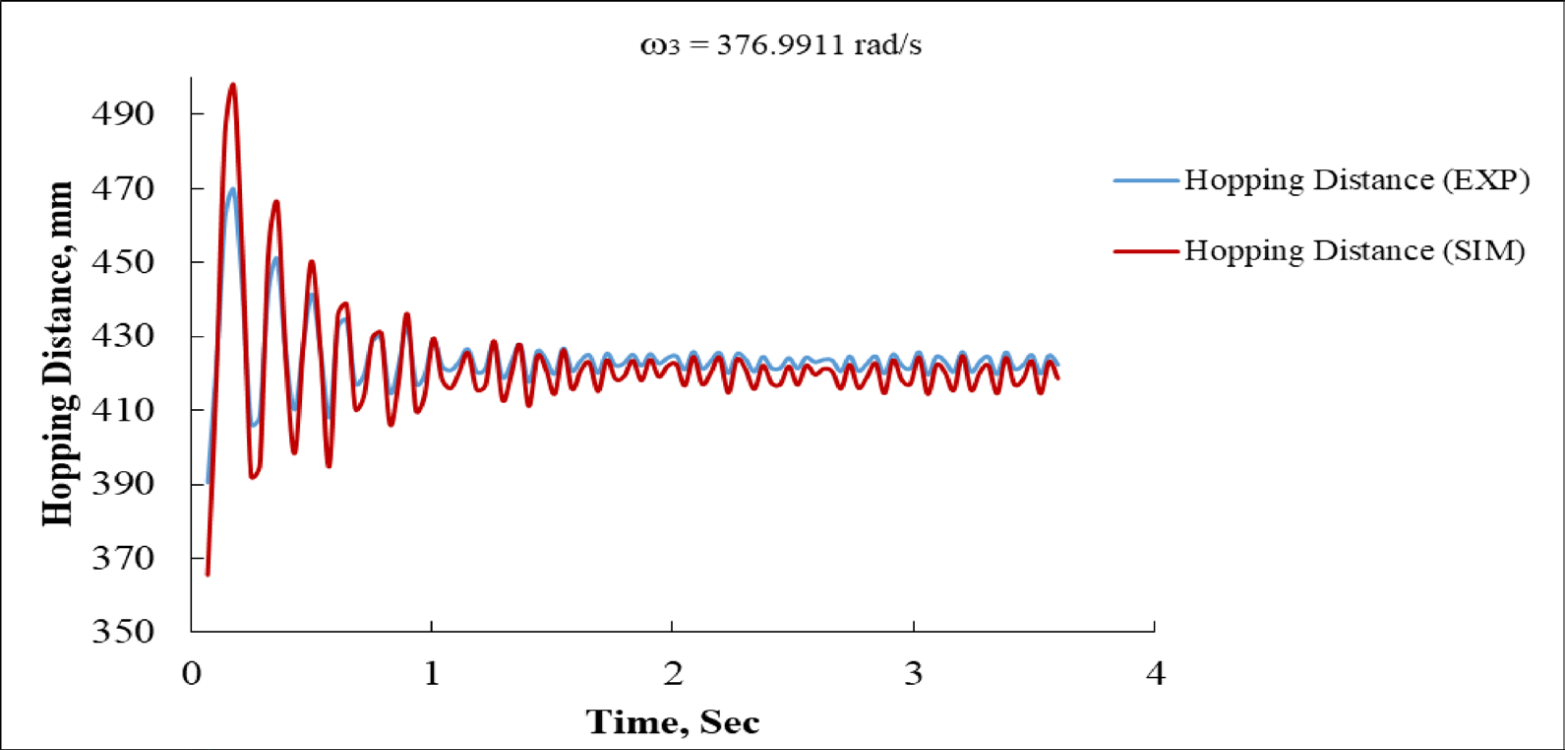
Hopping Distance vs. Time at ω_3_ = 376.9911 rad/s (EXP vs. SIM).

Figure 21 presents the hopping distance response, which clearly shows damped oscillations converging to a stable equilibrium value. The simulation predicts an initial peak hopping distance of nearly 490 mm, while the experiment records a slightly lower peak of approximately 470 mm. Both responses subsequently decrease through damped oscillations, with minima around 370 mm experimentally and 360 mm in the simulation. By 2.5 to 3 s, the responses converge and settle to a steady value of about 420 mm. The close overlap of the curves throughout the time domain confirms that the simulation effectively reproduces the experimental behavior for this parameter, with only slight overestimation of the initial peak, possibly due to un-modeled energy dissipation mechanisms such as air resistance or material damping.

While the simulation tends to slightly exaggerate initial oscillation amplitudes and smooth out fluctuations observed in the experimental data, the two approaches align closely in terms of long-term trends and final steady-state values. Specifically, the system stabilizes to a deviation near zero, achieves a body distance of about 950 mm after 4 s, maintains a steady oscillatory velocity around ± 500 mm/s, and stabilizes to a hopping distance of approximately 420 mm. Minor differences can be attributed to real-world effects such as friction, structural compliance, or sensor lag, which are not perfectly captured in the simulation. Overall, the results strongly validate the accuracy of the simulation model, with quantitative evidence demonstrating its ability to replicate experimental dynamics with high fidelity^2,36^.

**Fig. 22:**
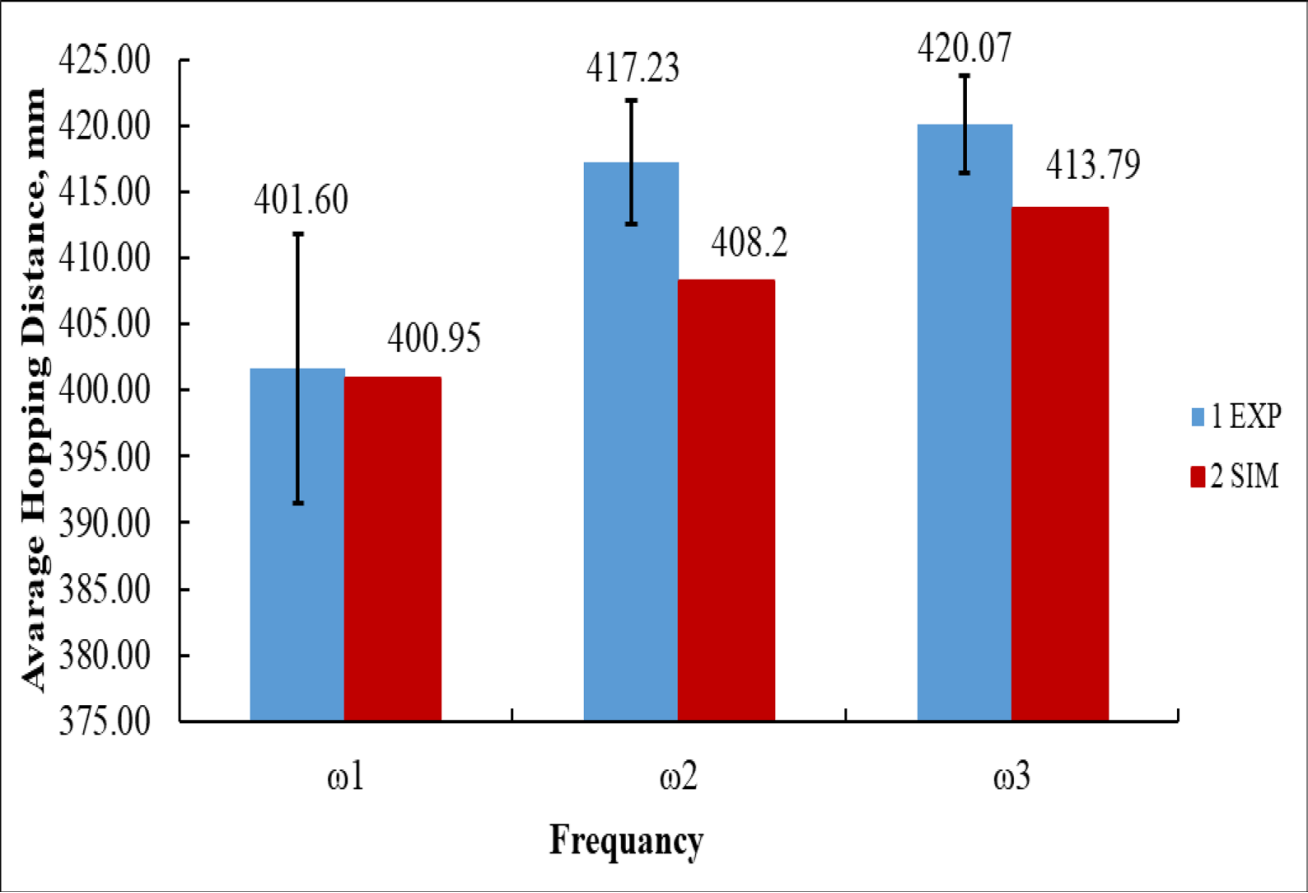
Average hopping distance at different frequencies: experimental vs. simulated results.

Figure 22 compares the average hopping distance of the vibration-driven U-shaped elastic beam robot obtained from experimental measurements (EXP) and simulation results (SIM) at three input angular velocities: ω₁ = 125.66 rad/s, ω₂ = 251.33 rad/s, and ω₃ = 376.99 rad/s.

At ω₁, the experimental average hopping distance was 401.60 mm, while the simulation predicted 400.95 mm. The difference between experiment and simulation was only 0.65 mm, showing excellent agreement at low frequency, though the experimental data showed a larger error margin (± 10 mm), indicating less stable performance^36^. At ω₂, the experimental result increased to 417.23 mm, while the simulation predicted 408.20 mm, giving a difference of 9.03 mm. Here, the experimental hopping was higher, but both trends confirmed improved locomotion with increased angular velocity^2^. At the highest angular velocity, ω₃, the experimental average hopping distance reached 420.07 mm, while the simulation result was 413.79 mm, corresponding to a difference of 6.28 mm.

Numerically, from ω₁ to ω₃, the experiment showed a total increase of 18.47 mm, whereas the simulation showed an increase of 12.84 mm. Both sets of results demonstrate a clear upward trend, confirming that higher angular velocities improve hopping performance. The figure also highlights that while simulation closely follows the experimental trend, it generally underestimates hopping distance by 2–9 mm, especially at medium and high input speeds. This indicates that the simulation successfully captures the vertical displacement dynamics but slightly misses the higher energy transfer observed in experiments^2,36^.

**Fig. 23:**
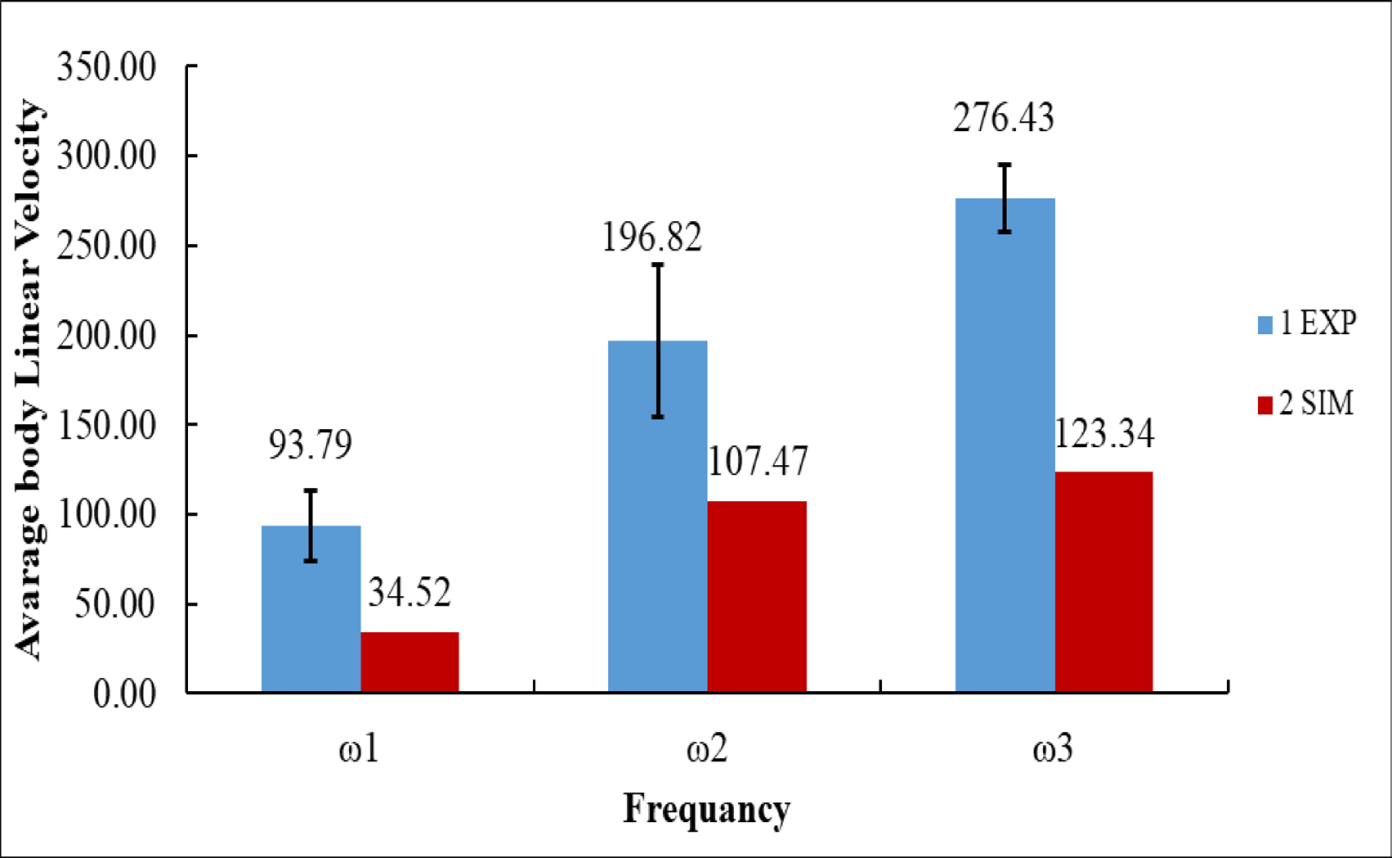
Average body linear velocity at different frequencies: experimental vs. simulated results.

Figure 23 presents a comparison between the experimental (EXP) and simulation (SIM) results of the robot’s average body linear velocity at three angular velocities: ω₁ = 125.66 rad/s, ω₂ = 251.33 rad/s, and ω₃ = 376.99 rad/s.

At the lowest speed, ω₁, the experimental measurement recorded an average body velocity of 93.79 mm/s, while the simulation predicted a much lower velocity of 34.52 mm/s. The difference of 59.27 mm/s indicates that the simulation significantly underestimates the robot’s forward motion at low frequency^16^. When the angular velocity increased to ω₂, the experimental velocity rose sharply to 196.82 mm/s^14^, nearly double that at ω₁. In contrast, the simulation produced a velocity of 107.47 mm/s, which, although closer to the experimental result, still underestimated it by 89.35 mm/s. At the maximum angular velocity, ω₃, the experimental velocity peaked at 276.43 mm/s, while the simulation output was 123.34 mm/s, resulting in a discrepancy of 153.09 mm/s^41^.

Numerically, the experimental results show a clear and consistent increase in body velocity, with gains of + 103.03 mm/s from ω₁ to ω₂ and + 79.61 mm/s from ω₂ to ω₃, for a total improvement of + 182.64 mm/s between ω₁ and ω₃. By comparison, the simulation also followed an increasing trend, with gains of + 72.95 mm/s (ω₁ to ω₂) and + 15.87 mm/s (ω₂ to ω₃), for a total rise of + 88.82 mm/s. However, across all cases, the simulation consistently underestimated body velocity by about 50–60%, which matches the limitation discussed in your manuscript^14,16^.

The hopping distance results indicate a robust correlation between experimental and simulated data, especially at elevated frequencies, but the body velocity comparison demonstrates a persistent underestimating by the simulation model. This underscores the necessity of modifying the simulation settings to more accurately reflect the dynamic performance of the actual system, especially regarding velocity generation^2^. The inconsistency indicates that although the vertical displacement mechanics are adequately predicted, the horizontal velocity response necessitates additional calibration to reconcile the simulation outcomes with experimental data.

## Conclusions

- This study details the design, modeling, simulation, and experimental validation of a vibration-driven terrestrial robot utilizing a U-shaped elastic beam actuated by a single DC motor with an eccentric rotating mass. The impact of angular velocity on locomotion performance, stability, and hopping behavior was rigorously investigated.
- Experimental findings indicated that increased angular velocities enhanced body velocity, hopping distance, and stability. Body velocity increased from 85.46 mm/s at ω₁ = 125.66 rad/s to 265.49 mm/s at ω₃ = 376.99 rad/s. In contrast, the hopping distance exhibited a more modest increase from 405.88 mm to 424.08 mm. Stability analysis demonstrated a significant reduction in deviation at elevated angular velocities, with the system stabilizing within ± 2 mm at ω₃.
- Simulation results accurately reflected the trend in hopping performance, with errors remaining below 3 mm at ω₃. The model consistently underestimated body velocity by 50–60% in all cases, indicating that friction, damping, and nonlinear stiffness effects were inadequately represented.
- The findings demonstrate that torsional vibrations in a U-shaped elastic beam facilitate locomotion through straightforward actuation methods, eliminating the need for complex control systems. The observed limitations, especially the discrepancy in velocity prediction, underscore the necessity for improved modeling of dissipative effects and nonlinear dynamics. Future research should concentrate on overcoming these limitations and investigating the scalability of this method to more intricate robotic systems or practical applications in restricted environments.

## Data Availability

The datasets used and/or analyzed during the current study are available from the corresponding author on reasonable request.
